# Ethnobotanical survey of the medicinal flora of Harighal, Azad Jammu & Kashmir, Pakistan

**DOI:** 10.1186/s13002-020-00417-w

**Published:** 2020-10-27

**Authors:** Muhammad Shoaib Amjad, Ujala Zahoor, Rainer W. Bussmann, Muhammad Altaf, Syed Mubashar Hussain Gardazi, Arshad Mehmood Abbasi

**Affiliations:** 1 Department of Botany, Women University of Azad Jammu & Kashmir, Bagh, 12500 Pakistan; 2 Department of Ethnobotany, Institute of Botany, Ilia State University, Tbilisi, Georgia; 3 Department of Zoology, Women University of Azad Jammu & Kashmir, Bagh, 12500 Pakistan; 4 Department of Environmental Sciences, COMSATS University Islamabad, Abbottabad Campus, Abbottabad, 22060 Pakistan

**Keywords:** Ethnobotany, Medicinal flora, Used value, Fidelity level, Azad Jammu & Kashmir, Pakistan

## Abstract

**Background:**

The present study is the first quantitative ethnobotanical evaluation of Harighal, an inaccessible and unexplored area of District Bagh Azad Jammu & Kashmir (AJK). The exploration, quantification, and comparison of ethnobotanical knowledge among different rural communities of the study area were mainly focused during field survey.

**Methodology:**

In total, 79 informants (49 men and 34 women) were selected randomly to collect data using a semi-structured questionnaire. Various quantitative indices, including use value, relative frequency of citation, relative importance, fidelity level, and informant consent factor, were employed to evaluate the gathered information. Furthermore, primary data were also compared with twenty-two papers published from adjoining areas.

**Result:**

A total of 150 medicinal plants belonging to 98 genera and 60 families were documented. Asteraceae, Fabaceae, and Rosaceae were the dominant families having 15 species each. Of these, 76 species were indigenous, 74 exotic, 136 were collected in the wild, 10 cultivated, and 4 both wild-collected and cultivated. Herbaceous taxa were the most used life form, and leaves were the most exploited plant part. Decoctions were the most preferred method used in preparation of herbal recipes. Three species viz. *Mentha longifolia*, *Berberis lycium*, and *Galium aparine* had the highest use value (1.05), relative frequency of citation (0.81), and relative importance value (96), respectively. The highest informant consensus factor (ICF) was reported for digestive disorders. *Mentha longifolia*, *Punica granatum*, *Zanthoxylum alatum*, and *Olea ferruginea* had 100% fidelity values. The Jaccard index revealed that uses of plants were more similar in two neighboring areas, i.e., Pearl Valley and Toli Peer.

**Conclusion:**

Local inhabitants still prioritize herbal medicines as an effective way to treat a wide variety of ailments. Elders and health practitioners of the study area are well aware of indigenous knowledge about medicinal plants, but young people are not much interested in herbal practices. Thus, valuable knowledge about the use of plants is on the verge of decline.

## Background

Ethnobotanical surveys focus on the complex connection between local inhabitants and local plants, including practices and cultural beliefs associated with different forms of uses [1–4]. These studies are important in highlighting the value of native plant species, e.g., for discovering novel drugs [5]. Medicinal plants are imperative for the livelihoods of underprivileged communities across the world [6–11]. Globally, 35,000–70,000 plant species are used in folk medicine [12]. In developing countries, 60–80% of the population is still relying on plant-based medicines because they are economical and safe alternative to often inaccessible allopathic medicine [13, 14]. Even in the developed world, herbal remedies are extensively used, e.g., 30–50% of the population in China, 40–50% in Germany, 48% in Australia, 42% in the USA, and 49% in France reported using herbal medicine as supplementary health care [15–17]. About 25% of modern allopathic drugs are derived directly from plants or synthetic analogues of different compounds isolated from medicinal plants [18]. Plant-based drugs are effective and often have less side effects. This can be best explained by comparison between the extract bark of important medicinal plant *Salix alba* (white willow) with the synthetic drug aspirin which has more reported side effects. Different studies confirmed that extract of *Salix alba* bark can avoid the side effects caused by aspirin [19].

The traditional knowledge of medicinal plants is held by many rural communities even in our times [20–24]. Such knowledge is transmitted from generation to generation [5, 25–27]. Differences and similarities in traditional knowledge and practice among two different cultural groups living within the same ecological region are fascinating, as they can provide understanding of how cultural reflection can change individual viewpoints about the environment and also guide interactions between human beings and resources in the ecosystem [28]. However, traditional knowledge on plant species is decreasing gradually across the globe [29]. This knowledge is usually held by hakims (traditional healers) and elderly people and be passed to the next generation via verbal communication only [30]; thus, there is a serious danger of knowledge loss due to the progression in the modern health care system, rapid urbanization, and poor relations between younger and old generation [31–34]. The documentation of traditional ethnomedicinal knowledge is of high importance and may contribute to the development of new drugs. Furthermore, this may also contribute to the maintenance of indigenous culture and natural resource management.

Pakistan has a large wealth of medicinal and aromatic plants due to its diverse habitat, climate, and soil types and harbors about 6000 wild plant species [35]. Among them, 400–600 species are used for therapeutic purposes. Eighty percent of this medicinal flora is restricted in Northwestern areas of Pakistan and Azad Kashmir [36–38]. In the early 1950s, 84% of population of Pakistan depended upon plants for treating various ailments; but nowadays, this practice is restricted to remote areas due to modernization and rapid change in lifestyle [39]. Previously, different ethnobotanical studies were conducted to document the traditional knowledge about medicinal plants and herbal recipes in remote areas of Pakistan and Azad Jammu & Kashmir [40–44]. However, Tehsil Harighal of District Bagh is still unexplored ethnobotanically, especially due to topographical challenges like hilly terrain and steep slopes, and cultural and religious restrictions that limits researcher access to document ethnobotanical knowledge. We hypothesized that due to the remoteness of the area, the ethnobotanical knowledge of Harighal would considerably differ from other areas of Pakistan. This study was planned with the objective to document the indigenous knowledge about medicinal plants used for primary health care particularly focusing on methods of preparation and administration of herbal recipes. The data was further analyzed by using various numerical indices and compared with previous studies to determine the novelty of work.

## Materials and methods

### Study area

**Fig. 1:**
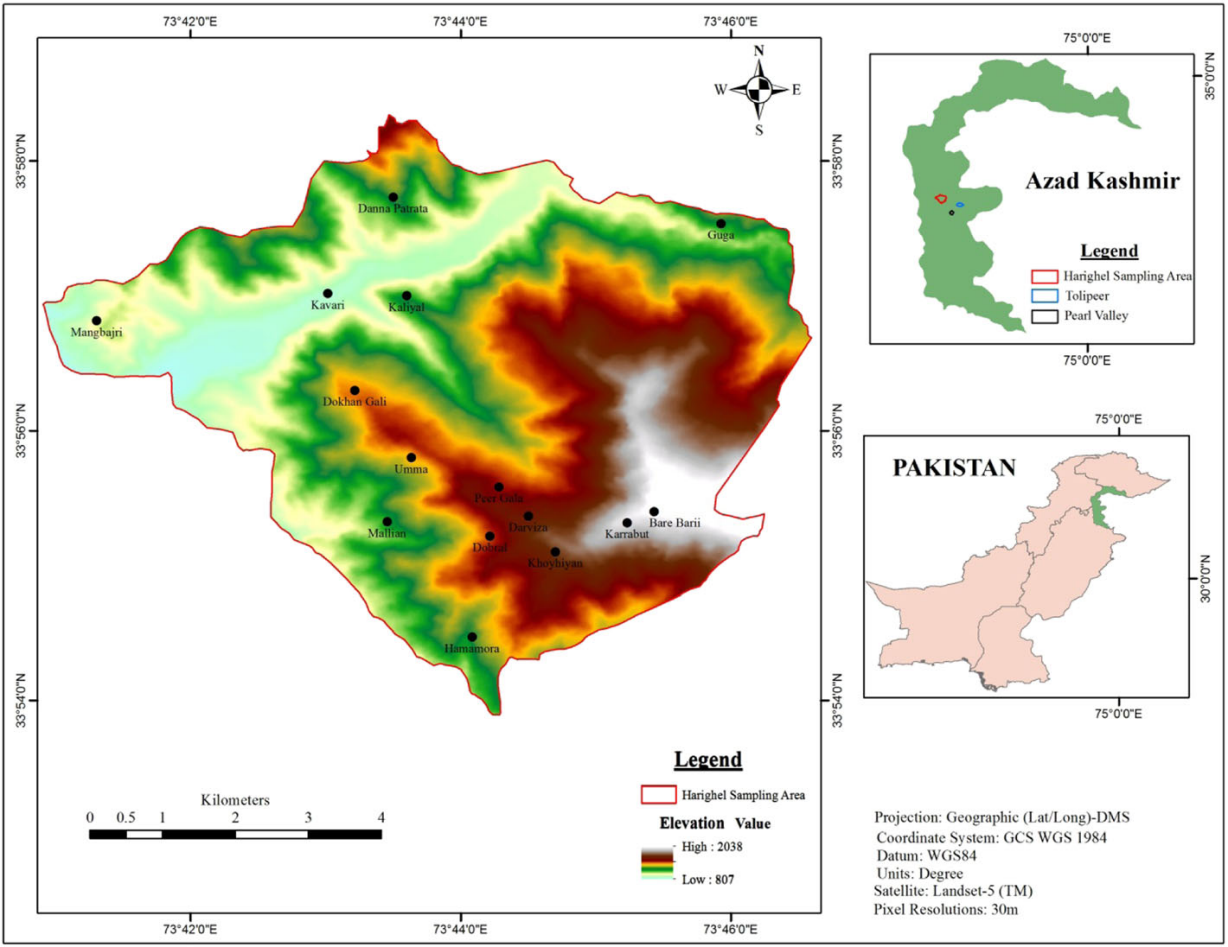
Map of the study area

**Fig. 2:**
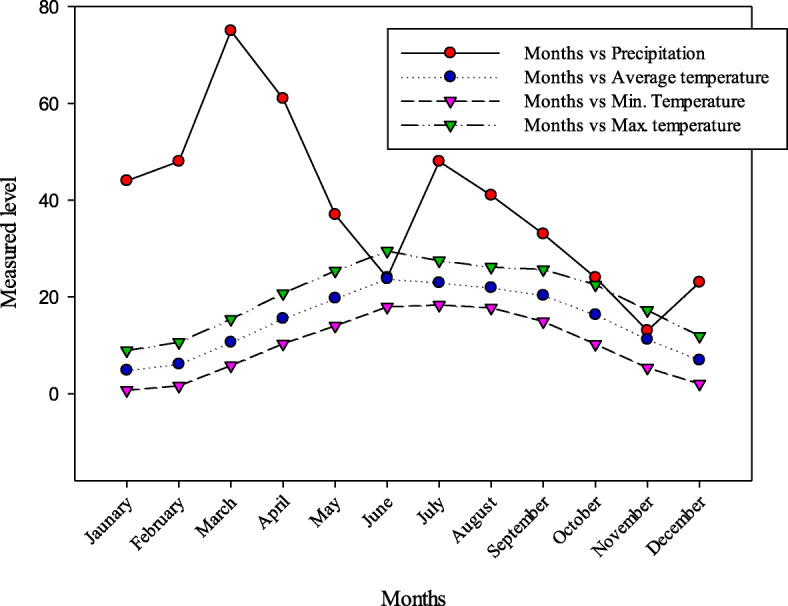
Precipitation and temperature of the study area

Harighal (33° 54′ 34° 08′ N to 73° 01′ 73° 38′ E), a Tehsil of District Bagh, is located in western Himalayan foothills of Pirpanjal, with altitude ranges between 900 and 2300 m (Fig. 1) [45]. It is 155 km away from Islamabad, the capital of Pakistan and 98 km away from the Muzaffarazad, the capital of Azad Jammu & Kashmir. The total area of Harighal is 712 km^2^ and its population is about 120,000 according to the 2017census. The climate is subtropical-temperate with about 1500 mm average annual precipitation. The summers are hot with temperature ranges between 21 and 40 °C while winters is cold with temperatures around 2 °C during January (Fig. 2) [46]. The vegetation is mainly dominated by *Olea ferruginea* at lower altitudes, *Pinus roxbughaii* and *Quercus incana* at mid altitudes, and *Pinus wallichiana* at higher altitude of the forest belt. Most of the area is occupied by open grassland.

The region has a diverse and complex ethnic composition with Rajputs, Maldial, Sudhan, Khawaja, Gujar, and Jat. Rajput tribes spread across the region and the Maldial tribe is regarded as the most influential ethnic group in Harighal, Azad Kashmir. Almost all of the inhabitants are Muslims. Popular languages are Pahari, Hindko, Gojri, and Kashmiri, but most inhabitants are also familiar with Urdu.

The area is remote, with difficult mountainous terrain and quite far from urban centers. Local inhabitants have poor socioeconomic conditions, lacking government services, and modern healthcare facilities. The roads and other infrastructure are poorly developed, and many inhabitants are dedicated to agriculture, livestock, and their own small-scale business. Some are educated and are government servants, while very few are serving abroad. Agriculture is mainly dependent upon rain-fed cropping system and maize is the main crop of the region. Basic health facilities are mainly provided by few public health dispensaries in the region, but locals residing at higher altitudes have very limited access to them and are mainly dependent upon herbal preparations for curing most ailments. Indigenous ethnomedicinal knowledge is mostly in the hand of elder people and health practitioners.

### Data collection

Ethnomedicinal data was collected from 79 informants including 45 men and 34 women during April 2017 to March 2018, using semistructured interviews and group discussions, after obtaining prior informed consent from the participants. Informants were selected randomly by convenience sampling (i.e., a sampling method in which units are selected based on easy access or availability) [47] and sometimes by using a snowball method [14]. Questionnaires were prepared according to Edward et al. [48]. The ethical guidelines provided by International Society of Ethnobiology (http://www.ethnobiology.net/) were strictly followed. The ethical approval to conduct the study was given before initiating surveys from the Ethics Committee of the Women University of Azad Jammu & Kashmir, Bagh. In addition to this, legal permission to conduct interviews was given by members of municipality committee. A prior informed consent form (PIC) was signed by all the informants after explaining the objective and consequence of study. The PIC was translated into local *Pahari* language. Sample size was determined by following Kadam and Bhalerao [49].

### Plant collection and identification

The medicinal plants were collected dried, pressed, and mounted on standard herbarium sheets following standard taxonomic methods [50]. The specimens were identified with the help of plant taxonomist and confirmed using flora of Pakistan (https://http://www.efloras.org) [51, 52]. Further verification of identified specimens was done at the Herbarium of Medicinal and Aromatic Plants in AJ&K established by the Pakistan Agriculture and Research Council (PARC). APG IV (2016) [53] was used for family taxonomy while The Plant List (2013) [54] was used to verify scientific names. The final voucher specimens were deposited in herbarium of the Women University of AJ&K, Bagh.

### Ethnobotanical indices

The homogeneity and validation of collected ethnomedicinal data was checked by applying following quantitative indices.

### Informant consensus factor

Emic use types were grouped in to 16 etic use categories following International Classification of Primary Care (ICPC) with some modification [55]. The agreement between the respondents about usage of plants for curing various groups of ailments was checked by informant consent factor. It was calculated by following Heinrich et al. [56] using given formula:

$$ Fic=\frac{Nur- Nt}{\left(\mathrm{Nur}-1\right)} $$

Where;

Nur = use-reports in selected group of diseases, Nt = species used for treating various diseases of that group. Informant consensus factor (ICF) values varied from 0 to 1, where value (close to 1) indicates that plant species are selected by using well-defined criteria or information and its uses are extremely exchanged among the informants and low values (close to 0) are obtained when plant species are chosen randomly or information about their use are not exchange among informants [20].

### Relative frequency of citation

The harmony between respondents on medicinal uses of plants in the study area was determined by relative frequency of citation (RFC). It was calculated by following Vijayakumar et al. [57] using the given formula:

$$ RFC= FC/N $$

Where;

FC = informants reporting use of a given species, *N* = total number of informants. This index is used to identify the most utilized/preferred plants in the area. FC value varies from 0 (when a plant species is not under any use in that area) to 1 (if all the informants refer plant species as useful). FC exhibits the local importance of each species without considering the use categories [31, 58].

### Use value index

The use value of plant species was determined by following Vijayakumar et al. [57] using the given formula:

$$ \mathrm{UV}=\frac{\Sigma \mathrm{Ui}}{\mathrm{N}\ } $$

Where;

Ui = use reports cited by each respondent for given species, *N* = total number of respondents. Use value reflects the relative importance of reported plant species in area. High use value shows that plant species have many use reports and is important in the region, whereas low use value (approach to 0) shows that species have few use reports related to its use. However, use report is not meaningful to differentiate whether a plant species is used for single or manifold purposes [20].

### Relative importance

It was calculated following Khan et al. [6] by using the given formula.

$$ RI=\left( RelPH+ RelBS\right)\times 100/2 $$

PH = pharmacological attribute of the selected plant, Rel PH = relative pharmacological attributes of a given plant.

$$ RelPH=\frac{\mathrm{PH}\ \mathrm{of}\ \mathrm{a}\ \mathrm{selected}\ \mathrm{plant}\ }{\mathrm{maximum}\ \mathrm{PH}\ \mathrm{of}\ \mathrm{a}\mathrm{ll}\kern0.5em \mathrm{plant}\ \mathrm{species}\ } $$

BS = body systems treated by selected plant species, Rel BS = relative body systems treated by selected species.

$$ RelBS=\frac{\mathrm{BS}\ \mathrm{of}\ \mathrm{a}\ \mathrm{given}\ \mathrm{plant}}{\mathrm{maximum}\ \mathrm{BS}\ \mathrm{of}\ \mathrm{a}\mathrm{ll}\ \mathrm{reported}\ \mathrm{plant}\ \mathrm{species}} $$

### Fidelity level

Fidelity level indicates the preference of particular plant species by informants to treat specific disease. It was calculated following Alexiades and Sheldon, [59] by using the given formula

$$ FL\%= Np/N\times 100 $$

Where;

Np = informants reporting use of particular plant species for a specific disease category, *N* = total number of informants who mentioned uses for a specific plant species for all disease category. High fidelity level (FL) value shows maximum frequency of use by the informants to treat a particular disease [20].

### Jaccard index

The similarity of knowledge between different communities was determined by comparing the findings of the current study with 22 published peer reviewed papers at regional, national, and global level by applying Jaccard index. These includes 9 studies from Azad Jammu & Kashmir, 7 form Khyber Paktunkhawa, and 3 from other areas of Pakistan. The studies conducted on the areas with similar, vegetation, climatic condition, and culture were consider for comparison. Further, the findings were also compared with 3 studies conducted in other developing counties including Nepal, India, and Ethopia. Jaccard index (JI) was calculated following Gonza et al. [60] by using the given formula:

$$ \mathrm{JI}=\frac{\mathrm{c}\times 100}{\left(\mathrm{a}+\mathrm{b}\right)-\mathrm{c}} $$

Where;

*a* = species of the study area, *b* = species of the neighboring area, and *c* = number of species common to both area.

## Results and discussion

### Demography and knowledge variation

A total of 79 informants were interviewed to collect medicinal plant knowledge based on their gender, age, and education (Table 1). The first category used for classification of informants was gender and 45 men and 34 women were interviewed. The easier availability and approachability to male informants and the prohibition of interaction of women with strangers, as well as and veiling (*parda*) forced us to interview more men than women. Demographic data demonstrates that women (average known species = 5.72; average cited uses = 9.38) had more knowledge about plants than men (average known species = 4.98; average cited uses = 8.05). Division of labor between genders in the area may be one reason for this difference, as men generally manage the fieldwork and earning, while women manage the indoor activities and domestic life, which are highly associated with herbal preparations to keep the family healthy. Similar findings were reported by other studies including Qaseem et al. [40] from Kotli, Ahmad et al. [44] from Neelum valley, and Kyani et al. [20] from Abbottabad. Age was used as second classification criterion and informants were classified into three major categories, i.e., above 60, between 40 and 60 and less than 40. Elders (age above 60) had more knowledge about plants than young people (age less than 40). Another reason for lower knowledge of young informants was their limited interest in herbal preparations due to changes in lifestyle with advent of industrialization and modernization. These findings were supported by other reports including Qaseem et al. [40] from Kotli and Umair et al. [61] from Hafizabad. Education was a third influential factor. Uneducated informants had a vast ethnobotanical knowledge, while tan educated informants had a more limited knowledge of plants. Likewise, traditional health practitioners had a broad traditional knowledge about medicinal uses of plants compared to other professions. Highly educated informants usually relied on allopathic medicines for their immediate healthcare, and had least knowledge about herbal medicines and their preparation methods. These finding are supported nationally by Kayani et al. [20], Yaseen et al. [22], and internationally by Giday et al. [62] and Tugume et al. [63].

**Table 1:** Demographic information of the Informants

Variables	IC	Number	ANSRI	ANURI
Gender	Men	45	4.98	8.05
	Women	34	5.72	9.38
	Total	79		
Age group	20-40	22	3.96	3.10
	41-60	46	8.40	4.96
	60-80	11	12.70	11.35
Education Level	Illiterate	26	5.95	4.08
	Elementary education	18	12.25	6.70
	Secondary education	13	11.90	6.11
	HSE	10	6.60	5.55
	Bachelor degree	7	6.15	5.01
	Higher education	5	10.80	6.71
Professions	THPs	13	25.55	13.64
	Midwives	10	13.2	10.43
	Herders	06	9.10	8.12
	Housewives	18	6.85	6.15
	Farmers	08	5.25	4.45
	Teachers	10	6.71	7.10
	Others	14	4.55	3.93

*IC* informants category, *ANSRI* average number of species reported by each informant, *ANURI* average number of use reported by each informant, *HSE* higher secondary education, *THPs* traditional health practionaires

### Diversity of ethnomedicinal flora

**Fig. 3:**
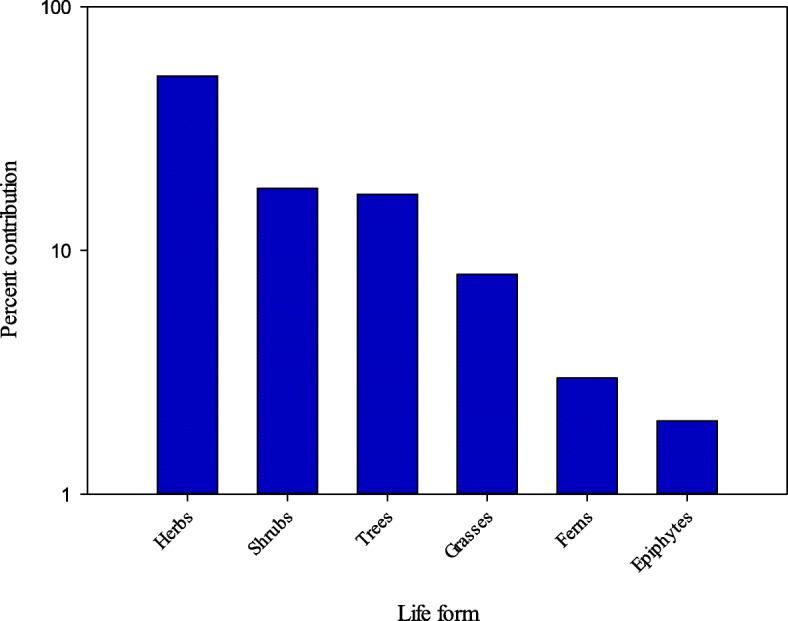
Life form distribution pattern of the reported plant species in the study area

A total of 150 medicinal plants belonging to 60 families and 98 genera were reported from study area (Table 2). Out of total 150 species, 76 were endemic or native and 74 exotic and among them, 136 plant species were wild, 10 were cultivated, and 4 were both wild-collected and cultivated (Table 1S). The herbaceous life form was dominantly (78 sp.; 52%) used in herbal preparation followed by shrubs (27 sp.; 18%), trees (25 sp.; 16.6 %), grasses (12 sp.; 8%), ferns (5 sp.; 3.3%), and epiphytes or climbers (Fig. 3). These findings are in accordance with previous reports [40, 41, 79, 80]. The predominance of the herbaceous habit in mountainous areas is a common ecological phenomena throughout the world [17, 44, 81]. The reason might be the high rainfall and moisture content at higher altitudinal areas [20, 41, 47].

**Table 2:** Medicinal uses of the reported taxa and their comparison with previous reports

Scientific name/voucher number/habit	Local name	Part used	Method of preparation/mode of application	Diseases treated	Previous use reports
Acanthaceae					
Acanthaceae					
*Dicliptera bupleuroides* Nees in Wall./UZ-02/H	Somni	Lf	pas, ext dec, int	Wounds Cough, **Diabetes**	1◊, 2◊, 3©, 4©, 5◊, 6◊, 7◊, 8◊, 9◊, 10◊, 11◊, 12◊, 13◊, 14◊, 15◊, 16◊, 17◊, 18◊, 19◊, 20◊, 21◊, 22◊
*Justicia adhatoda* L./UZ-31/S	Baikher	Bk Lf Rt	pow, int pow, int pow, int	**Stomachache,** Constipation Asthma Cough	1◊, 2◊, 3◊, 4©, 5◊, 6◊, 7◊, 8◊, 9◊, 10◊, 11◊, 12◊, 13◊, 14◊, 15◊, 16◊, 17◊, 18©, 19◊, 20◊, 21◊, 22◊
Amaranthaceae					
*Achyranthes aspera* L./UZ-90/ H	Puthcanda	Wp Lf Rt	pas, ext jui, int dec, int	Scorpion stings and Snake bites **Eye diseases** Inflammation	1, 2, 3, 4, 5, 6▲, 7◊, 8▲, 9©, 10◊, 11◊, 12◊, 13◊, 14◊, 15◊, 16◊, 17◊, 18©, 19◊, 20◊, 21▲, 22◊
*Alternanthera pungens* Kunth/UZ-79/H	Khaki buti	Lf	dec, int	Skin Infection, **Cuts and external injury**	1◊, 2◊, 3◊, 4◊, 5◊, 6◊, 7◊, 8◊, 9◊, 10◊, 11◊, 12◊, 13◊, 14◊, 15◊, 16◊, 17◊, 18◊, 19◊, 20◊, 21◊, 22◊
*Amaranthus spinosus* L./UZ-71/H	Jungli Ganayar	Lf Lf	dec, int Pas, int	Biliousness, Eye infection **Constipation**	1◊, 2▲, 3◊, 4◊, 5◊, 6▲, 7◊, 8◊, 9◊, 10◊, 11◊, 12◊, 13◊, 14◊, 15◊, 16◊, 17◊, 18◊, 19◊, 20◊, 21▲, 22◊
*Amaranthus viridis* L./UZ-50/H	Ganyar	Wp Lf	pow, int Pas, ext	Diarrhea, **Malaria**, Jaundice Antidote against snake and spider bites	1▲, 2▲, 3◊, 4◊, 5◊, 6▲, 7◊, 8▲, 9◊, 10◊, 11◊, 12◊, 13▲, 14◊, 15◊, 16◊, 17◊, 18▲, 19◊, 20◊, 21▲, 22▲
Amaryllidaceae					
*Allium griffithianum* Boiss./UZ-42/H	Piazi	Ae	coo, int	**Dyspepsia**, Flatulence	1◊, 2◊, 3©, 4©, 5◊, 6◊, 7◊, 8◊, 9◊, 10◊, 11◊, 12◊, 13◊, 14◊, 15◊, 16◊, 17◊, 18◊, 19◊, 20◊, 21◊, 22◊
Apiaceae					
*Angelica glauca* Edgew./UZ-101/H	Choora	Rt	inf, Int	**Fever**, Colds	1◊, 2◊, 3◊, 4◊, 5▲, 6◊, 7◊, 8◊, 9◊, 10◊, 11◊, 12◊, 13▲, 14◊, 15◊, 16◊, 17◊, 18◊, 19◊, 20◊, 21◊, 22◊
*Torilis japonica* (Houtt.) DC./UZ-69/H	Lahndara	Sd Rt	pow, int dec, int	Skin diseases, **Indigestion**	1◊, 2◊, 3◊, 4◊, 5◊, 6◊, 7◊, 8◊,9◊,10◊,11◊,12◊,13◊,14◊,15◊,16◊,17◊,18◊,19◊,20◊,21◊,22◊
Apocynaceae					
*Carissa opaca* Stapf. ex. Haines./UZ-110/S	Garanda	Wp	pow, int	Joint pain, Scabies, **Jaundice**, Inflammation	1◊, 2◊, 3◊, 4©, 5◊, 6▲, 7◊, 8◊, 9◊, 10◊, 11◊, 12◊, 13◊, 14◊, 15◊, 16◊, 17◊, 18©, 19◊, 20◊, 21◊, 22◊
*Nerium oleander* L./UZ-122/S	Kanair	Fl Lf	jui, int jui, int	Cough **Flu, Fever**, Toothache, Blood pressure, Antidote	1◊, 2◊, 3▲, 4▲, 5◊, 6▲, 7◊,8◊,9◊,10◊,11◊,12◊,13◊,14◊,15◊,16◊,17◊,18◊,19▲,20◊,21▲,22◊
Araliaceae					
*Hedera nepalensis* K. Koch./UZ-147/H	Bail	Lf	dec, int	Nervous system disorders, **Rheumatism**, Abnormal sweating	1◊, 2▲, 3▲, 4▲, 5◊, 6©, 7▲, 8◊,9◊,10◊,11▲,12◊,,13◊,14◊,15◊,16◊,17◊,18◊,19◊,20◊,21◊,22◊
Asclepiadaceae					
*Vincetoxicum hirundinaria* Medik./UZ-131/H	Medhshingi	Ae	dec, int	Boils, **Pimples**	1◊, 2◊, 3©, 4©, 5◊, 6◊, 7◊, 8◊,9◊,10◊,11◊,12◊,13◊,14◊,15◊,16◊,17◊,18◊,19◊,20◊,21◊,22◊
Aspleniaceae					
*Asplenium dalhousiae* Hook./UZ-108/F	Gutti	Wp	dec, int	**Typhoid**	1◊, 2◊, 3◊, 4◊, 5◊, 6◊, 7◊, 8◊,9◊,10◊,11◊,12◊,13◊,14◊,15◊,16◊,17◊,18◊,19◊,20◊,21◊,22◊
Asteraceae					
*Achillea millefolium* L./UZ-117/H	kangi.i	Fl Lf	pas, int dec, int	**Digestive problems**, Brain tonic, Female organ problems Colds and Fever	1◊, 2◊, 3◊, 4◊, 5▲, 6▲, 7©, 8◊,,9◊,10◊,11◊,12◊,13▲,14◊,15◊,16◊,17◊,18◊,19◊,20©,21◊,22◊
*Anaphalis adnata* Wall. ex DC.*/*UZ-111/H	Dialect	Lf	pow, ext	Bleeding, **Wound healing**	1◊, 2◊, 3◊, 4©, 5◊, 6◊, 7◊, 8◊,9◊,10◊,11◊,12◊,13◊,14◊,15◊,16◊,17◊,18◊,19◊,20◊,21◊,22◊
*Artemisia vulgaris* L./UZ-20/H	Chaow	Lf	jui, int	**Kill worms,** Skin diseases	1◊, 2©, 3◊, 4◊, 5◊, 6◊, 7◊, 8◊,9◊,10◊,11◊,12◊,13◊,14◊,15◊,16◊,17◊,18◊,19◊,20◊,21◊,22◊
*Bidens biternata* (Lour.) Merr. & Sherff./UZ-74/H	Palouthi	Lf Rt	jui, int pas, ext	**Sore throat** Toothache	1◊, 2▲, 3◊, 4◊, 5◊, 6©,7◊, 8◊,9◊,10◊,11◊,12◊,13◊,14◊,15◊,16◊,17◊,18◊,19◊,20◊,21◊,22◊
*Cirsium vulgare* (Savi) Ten*.* /UZ-32/H	Kandiara	Rt Wp Wp	pow, int ash, int Inf, int	**Sore jaws** Rheumatic joints Bleeding piles	1◊, 2◊, 3◊, 4◊, 5◊, 6◊, 7◊, 8◊,9◊,10◊,11◊,12◊,13◊,14◊,15◊,16◊,17◊,18◊,19◊,20◊,21◊,22◊
*Conyza Canadensis* (L.) Cronquist/UZ-01/H	Kali Buti	Lf	dec, int	Scanty Urination, **Dysentery,** Diarrhea, Hemorrhages	1◊, 2©, 3◊, 4,◊ 5▲, 6◊, 7◊, 8◊,9◊,10◊,11◊,12◊,13◊,14◊,15◊,16◊,17◊,18◊,19◊,20◊,21◊,22◊
*Crepis multicaulis* Ledeb./UZ-11/H	Shina	Fl	pas, ext	**Eye infection**	1◊, 2◊, 3◊, 4◊, 5◊, 6◊, 7◊, 8◊,,9◊,10◊,11◊,12◊,13◊,14◊,15◊,16◊17◊,18◊,19◊,20◊,21◊,22◊
*Gerbera gossypina* (Royle) Beauverd/UZ-129/H	Put putiola	Lf	pas, ext	Bone fractures, Wounds, Cuts, Pain, **Skin diseases**	1◊, 2◊, 3▲, 4©, 5◊, 6◊, 7◊, 8◊,9◊,10◊,11◊,12◊,13◊,14◊,15◊,16◊,17◊,18◊,19◊,20◊,21◊,22◊
*Helianthus annuus* L*./*UZ-104/H	Souraj mukhi	Fl Sd Lf	pas, ext eat, int ext, int	Skin diseases Scanty urination, **Curing chest infections,** Liver ailments Lung ailments, Piles, Kinney problems	1◊, 2◊, 3◊, 4◊, 5◊, 6◊, 7◊, 8◊,9◊,10◊,11◊,12◊,13◊,14◊,15◊,16◊,17◊,18◊,19◊,20◊,21◊,22◊
*Launaea procumbens* (Roxb.) Ram. & Raj./UZ-41/H	Hund	Wp	rfo, Int	Diabetes, **Pain**	1◊, 2◊, 3◊, 4◊, 5◊, 6◊, 7◊, 8▲,9◊,10◊,11◊,12◊,13◊,14◊,15◊,16◊,17◊,18◊,19◊,20◊,21▲,22◊
*Silybum marianum* (L.) Gaertn./UZ-52/H	Kandiyar	Lf	exr, int	Liver problems, Scanty urination, **Stomachic**, Tonic, Respiratory tract infection	1◊, 2◊, 3◊, 4◊, 5◊, 6◊, 7◊, 8◊,9◊,10◊,11◊,12◊,13◊,14◊,15◊,16▲,17◊,18◊,19◊,20◊,21◊,22◊
*Sonchus oleraceus* (L.) L./ UZ-62/H	Dodak	Lf	coo, int	**Abdominal pain**	1◊, 2◊, 3◊, 4◊, 5◊, 6◊, 7◊, 8◊,9◊,10◊,11◊,12◊,13◊,14◊,15◊,16◊,17◊,18◊,19◊,20◊,21◊,22◊
*Tagetes minuta* L*.*/UZ-150/H	Sadberga	Lf	jui, int	**Earache**	1◊, 2◊, 3◊, 4◊, 5◊, 6◊, 7◊, 8◊,,9◊,10◊,11◊,12◊,13◊,14◊,15◊,16◊,17◊,18▲,19◊,20◊,21◊,22◊
*Taraxacum officinale (*L.) Weber ex F.H.Wigg./UZ-93/H	Hend	Rt	jui, int	**Scanty urination,** Liver tonic, Diabetes	1©, 2©, 3©, 4©, 5▲, 6©, 7◊, 8◊,9▲,10◊,11◊,12▲,13◊,14◊,15◊,16◊,17◊,18▲,19◊,20▲,21◊,22▲
*Xanthium strumarium* L./UZ-81/S	Souriyala	Lf Fr	jui, int pow, int	Chronic mild fever Cooling, Infections, **Urinary problems**	1 ◊, 2©, 3 ◊, 4 ◊, 5 ◊, 6 ◊, 7 ◊, 8▲,9▲,10▲,11◊,12◊,13◊,14◊,15◊,16◊,17◊,18◊,19◊,20◊,21▲,22▲
Asparagaceae					
*Polygonatum geminiflorum* Decne./ UZ- 146/H	Noorialam	Lf	inf, int	Treat pain, **Fever**, Inflammation, Allergy, weakness	1◊, 2◊, 3◊, 4◊, 5◊, 6◊, 7◊, 8◊,9◊,10◊,11◊,12◊,13◊,14◊,15◊,16◊,17◊,18◊,19◊,20◊,21◊,22◊
Balsaminaceae					
*Impatiens edgeworthii* Hook*.*f./UZ-21/H	Batmandar/ buntil	Wp Wp	exr, int past, ext	Urinary tract infection, **Fever** Burns	1◊, 2◊, 3◊, 4◊, 5◊, 6◊, 7◊, 8◊,9◊,10◊,11◊,12▲,13◊,14◊,15◊,16◊,17◊,18◊,19◊,20◊,21◊,22◊
Berberidaceae					
*Berberis lycium* Royle/UZ-12/S	Sumbal	Fr Bk	eat, int dec, int	**Cough** Skin problems, Liver problems	1▲, 2©, 3▲, 4▲, 5▲, 6▲, 7▲, 8◊,9◊,10▲,11◊,12◊,13▲,14◊,15◊,16◊,17◊,18▲,19▲,20▲,21◊,22▲
Boraginaceae					
*Cynoglossum lanceolatum* Forssk./UZ-140/H	Churoun	Rt	exr, int	**Throat ache**	1◊, 2◊, 3◊, 4◊, 5◊, 6▲, 7◊, 8◊,9◊,10◊,11◊,12▲,13◊,14◊,15◊,16◊,17◊,18◊,19◊,20▲,21◊,22◊
*Trichodesma indicum* (L.) Lehm./UZ-03/H	Handusibooti	Fl Lf / Rt	fra, int exr, int	Brain refreshment **Scanty urination**, Blood purifier	1◊, 2◊, 3◊, 4▲, 5▲, 6©, 7◊, 8◊,9▲,10◊,11◊,12◊,13◊,14◊,15◊,16◊,17◊,18▲,19◊,20◊,21▲,22◊
Brassicaceae					
*Capsella bursa-pastoris* (L.) Medic./UZ-33/H	Saag	Wp Sd Sd	eat, int pow, int flu, Int	**Chest infections** Bleeding	1◊, 2◊, 3◊, 4◊, 5◊, 6▲, 7◊, 8©,9◊,10◊,11◊,12◊,13©,14◊,15◊,16◊,17◊,18◊,19◊,20◊,21◊,22◊
Buxaceae					
*Sarcococca saligna* (D. Don) Muel /UZ-43/H	Ladan/ bansathra	Lf Rt	dec, int jui, int	**Joint pain,** Dysentery Gonorrhea	1◊, 2▲, 3©, 4©, 5◊, 6◊, 7◊, 8◊,9◊,10◊,11◊,12◊,13◊,14◊,15◊,16◊,17◊,18◊,19◊,20◊,21◊,22◊
Cannabaceae					
*Cannabis sativa* L./UZ-54/H	Bhang	Lf	pow, int	**Astringent that bowels stomachic,** Leprosy, Tonic, Narcotic action	1▲, 2◊, 3◊, 4◊, 5▲, 6◊, 7▲, 8◊,9◊,10▲,11▲,12◊,13▲,14◊,15◊,16◊,17▲,18▲,19▲,20▲,21▲,22▲
Caprifoliaceae					
*Viburnum grandiflorum* Wall. ex DC./UZ-72/S	Guch	Sd	jui, int	**Typhoid**, Whooping cough	1▲, 2◊, 3©, 4©, 5◊, 6◊, 7◊, 8◊,9◊,10▲,11◊,12◊,13▲,14◊,15◊,16◊,17◊,18◊,19◊,20◊,21◊,22◊
Celastraceae					
*Maytenus nemorosa* Marais /UZ-82/S	Patakhi	Wp	ext, ext	**Toothache,** Eye inflammation	1◊, 2◊, 3◊, 4◊, 5◊, 6◊, 7◊, 8◊,9◊,10◊,11◊,12◊,13◊,14◊,15◊,16◊,17◊,18◊,19◊,20◊,21◊,22◊
Chenopodiaceae					
*Chenopodium album* L./UZ-61/H	SkhaBotey	Wp	dec, ext	**Skin diseases**	1▲, 2▲, 3◊, 4◊, 5◊, 6▲, 7◊, 8©,9◊,10◊,11▲,12▲,13▲,14◊,15▲,16◊,17◊,18▲,19▲,20◊,21▲,22▲
Convolvulaceae					
*Convolvulus arvensis* L./UZ-103/C	Hirrankhuri	Wp	ext, int	Piles, Dandruff, **Constipation**	1◊, 2◊, 3◊, 4◊, 5◊, 6◊, 7◊, 8▲,9◊,10◊,11◊,12◊,13◊,14▲,15◊,16◊,17▲,18◊,19▲,20◊,21▲,22◊
*Ipomoea purpurea* (L.) Roth /UZ-2/H	Bahrwa	Wp Sd	pou, Int pow, Int	**Skin disease**, Cancer Tonic, constipation	1◊, 2▲, 3◊, 4◊, 5◊, 6◊, 7◊, 8◊,9◊,10◊,11◊,12◊,13◊,14◊,15◊,16◊,17◊,18◊,19◊,20◊,21◊,22◊
Commelinaceae					
*Commelina benghalensis* L./UZ-128/S	Kanchara	Wp	pou, Ext	**Skin diseases**	1◊, 2◊, 3◊, 4◊, 5◊, 6©, 7◊, 8◊,9◊,10◊,11◊,12◊,13◊,14◊,15◊,16◊,17◊,18◊,19◊,20◊,21◊,22◊
Cornaceae					
*Cornus macrophylla* Wall./UZ-133/T	Kandar	Bk	pow, int	**Backache**	1◊, 2©, 3◊, 4◊, 5◊, 6◊, 7◊, 8◊,9◊,10◊,11◊,12◊,13◊,14◊,15◊,16◊,17◊,18◊,19◊,20◊,21◊,22◊
Cuscutaceae					
*Cuscuta reflexa* Roxb.*/*UZ-12/H	Neeladari	Wp	jui, int	Jaundice, **Dandruff**	1◊, 2◊, 3◊, 4©, 5◊, 6◊, 7◊, 8◊,9▲,10◊,11◊,12◊,13◊,14◊,15◊,16◊,17◊,18©,19◊,20◊,21▲,22◊
Cyperaceae					
*Cyperus rotundus* L./UZ-145/H	Kah	Rt	mix, int	Dysentery, Gastric problems, **Intestinal disorders**	1◊, 2▲, 3◊, 4◊, 5◊, 6▲, 7◊, 8▲,9▲,10◊,11◊,12◊,13▲,14◊,15◊,16◊,17◊,18◊,19◊,20◊,21▲,22◊
Dryopteridaceae					
*Dryopteris filix-mas* (L.) Schoot/UZ-120/F	Kungi	Fd	veg, int	**Constipation**	1◊, 2◊, 3◊, 4◊, 5◊, 6◊, 7◊, 8◊,9◊,10◊,11◊,12◊,13◊,14◊,15◊,16◊,17◊,18◊,19◊,20◊,21◊,22◊
Elaeagnaceae					
*Elaeagnus umbellata* Thunb./UZ-22/S	Kankolii	Sd Ol Fl	pow, int exr, int dec, int	Stimulant in Cough **Pulmonary infections** Cardiac problems	1◊, 2◊, 3©, 4©, 5◊, 6◊, 7◊, 8◊,9◊,10◊,11◊,12◊,13◊,14◊,15◊,16◊,17◊,18◊,19◊,20◊,21◊,22◊
Euphorbiaceae					
*Euphorbia helioscopia* L./UZ-13/H	Dhoudhal	Wp Wp Wp	ert, ext pow, int lat, Int	Kill worms **Healing wounds** Poisonous effect that cause swelling in skin	1◊, 2◊, 3▲, 4▲, 5▲, 6▲, 7◊, 8▲,9▲,10◊,11◊,12◊,13▲,14◊,15◊,16◊,17◊,18▲,19◊,20◊,21▲,22◊
*Ricinus communis* L./UZ-04/S	Arind	Sd Lf	oil, int dec, ext	Constipation Skin diseases, Joint pain, **Muscles swelling**, Eye infection	1◊, 2▲, 3◊, 4◊, 5◊, 6, 7◊, 8◊, 9◊, 10◊, 11◊, 12◊, 13◊, 14◊, 15◊, 16◊, 17▲, 18©, 19◊, 20◊, 21◊, 22◊
Fabaceae					
*Acacia modesta* Wall*./*UZ-63/T	Kikar	Br	rfo, int	Anti-inflammatory, **Toothache**	1◊, 2◊, 3◊, 4◊, 5◊, 6▲, 7◊, 8◊,9◊,10◊,11◊,12◊,13◊,14◊,15◊,16◊,17▲,18◊,19▲,20◊,21▲,22◊
*Acacia nilotica* (L.) Delile/UZ-84/T	Desi kikar	Bk Pd	pas, int pow, ext	Piles, Dysentery, Diarrhea **Anti-dandruff**	1◊, 2◊, 3◊, 4◊, 5◊, 6◊, 7◊, 8◊,9▲,10◊,11◊,12◊,13◊,14◊,15◊,16◊,17◊,18◊,19◊,20▲,21▲,22◊
*Astragalus canadensis* L./UZ-125/H	Tindni	Lf	inf, int	**Stomachache**	1◊, 2◊, 3◊, 4◊, 5◊, 6◊, 7◊, 8◊9◊,10◊,11◊,12◊,13◊,14◊,15◊,16◊,17◊,18◊,19◊,20◊,21◊,22◊
*Bauhinia variegata* L./UZ-14/T	Kachnar	Fl	inf, int	Tonic, **Gastric problems**	1◊, 2◊, 3◊, 4◊, 5◊, 6◊, 7◊, 8◊,9◊,10◊,11◊,12◊,13◊,14◊,15◊,16◊,17◊,18◊,19◊,20◊,21◊,22◊
*Crotalaria juncea* L./UZ-95/H	Sunn	Sd Rt	pas, ext exr, int	**Hair tonic,** Skin diseases Colic, Epistaxis	1◊, 2◊, 3◊, 4◊, 5◊, 6◊, 7◊, 8◊,9◊,10◊,11◊,12◊,13◊,14◊,15◊,16◊,17◊,18◊,19◊,20◊,21◊,22◊
*Dalbergia sissoo* DC./UZ-55/T	Shishm	Lf	dec, int	Eye pain, Body pain, Diarrhea, Jaundice, **Abdominal pain,** Skin diseases	1◊, 2◊, 3◊, 4◊, 5◊, 6©, 7◊, 8◊,9◊,10◊,11◊,12◊,13◊,14◊,15◊,16◊,17◊,18▲,19◊,20◊,21▲,22◊
*Desmodium elegans* DC./UZ-142/S	Halphaat	Rt	tea, int	Hypertension, **Antidote**	1◊, 2▲, 3◊, 4◊, 5◊, 6◊, 7◊, 8◊,9◊,10◊,11◊,12◊,13◊,14◊,15◊,16◊,17◊,18◊,19◊,20◊,21◊,22◊
*Indigofera linifolia* (L. f.) Retz./UZ-46/H	Jund	Lf	rfo, ext	**Skin allergy**	1◊, 2◊, 3◊, 4◊, 5◊, 6, 7◊, 8◊,9◊,10◊,11◊,12◊,13◊,14◊,15◊,16◊,17◊,18◊,19◊,20◊,21©,22◊
*Lathyrus aphaca* L./UZ-136/H	Jangli matter	Sd	pow, int	**Narcotic**	1◊, 2◊, 3◊, 4◊, 5◊, 6◊, 7◊, 8©, 9◊, 10◊, 11◊, 12◊, 13◊, 14◊, 15◊, 16◊, 17◊, 18◊, 19◊, 20◊, 21◊, 22◊
*Lespedeza juncea* (L.f.) Pers./UZ-78/H	Kuchani	Rt	jui, int	Diarrhea, **Dysentery**	1◊, 2▲, 3©, 4©, 5◊, 6◊, 7◊, 8◊,9◊,10◊,11◊,12◊,13◊,14◊,15◊,16◊,17◊,18◊,19◊,20◊,21◊,22◊
*Lotus corniculatus* L*./*UZ-105/H	Sriri	Fl Rt Wp	pou, Int exr, int pou, ext	Cardiotonic, **Sleeping problems** Gastric problems Tonic Skin inflammation	1◊, 2◊, 3◊, 4◊, 5◊, 6◊, 7◊, 8◊,9◊,10◊,11◊,12◊,13◊,14◊,15◊,16◊,17◊,18◊,19◊,20◊,21◊,22◊
*Medicago polymorpha* L./UZ-66/H	Maina	Lf	exr, int	**Dysentery**, Indigestion	1◊, 2◊, 3◊, 4◊, 5◊, 6◊, 7◊, 8◊,9◊,10◊,11◊,12◊,13◊,14◊,15◊,16◊,17◊,18▲,19▲,20◊,21◊,22◊
*Mimosa pudica* L./UZ-118/H	Choi Moi	Lf	dec, int	Hemorrhoids, Urinary infections, Cancer, **Diabetes**, , Hepatitis, Obesity, Sores, Piles, Glandular swellings	1◊, 2◊, 3◊, 4◊, 5◊, 6◊, 7◊, 8◊,9◊,10◊,11◊,12◊,13◊,14◊,15◊,16◊,17◊,18◊,19◊,20◊,21◊,22◊
*Trifolium resupinatum* L./UZ-85/H	Tilpetra	Sd	che, int	Digestive disorder like **Abdominal pain.**	1◊, 2◊, 3◊, 4◊, 5◊, 6◊, 7◊, 8©,9◊,10◊,11◊,12◊,13◊,14◊,15◊,16◊,17◊,18◊,19◊,20◊,21◊,22◊
*Vicia sativa* L./UZ-25/C	Phalli	Wp	inf, int	Indigestion, Tonic, Scanty urination, **Asthma,** Cough, Bronchitis, Skin diseases, Urinary problems	1◊, 2◊, 3◊, 4◊, 5◊, 6▲, 7◊, 8©,9◊,10◊,11◊,12◊,13◊,14◊,15◊,16◊,17◊,18◊,19◊,20◊,21◊,22◊
Geraniaceae					
*Geranium rotundifolium* L.*/*UZ-34/H	Rattan joge	Rt	inf, int	**Mouth ulcers,** Stomach ulcer, Hemorrhoids	1◊, 2◊, 3◊, 4◊, 5◊, 6◊, 7◊, 8◊,9◊,10◊,11◊,12◊,13◊,14◊,15◊,16◊,17◊,18◊,19◊,20◊,21◊,22◊
Gentianaceae					
*Swertia cordata* (Wall. ex G. Don) C.B. Clarke/UZ-44/H	Cheratbotay	Ae	exr, int	Digestive problem, Liver problems, **Diabetes,** Nausea	1◊, 2▲, 3◊, 4◊, 5◊, 6◊, 7◊, 8◊,9◊,10◊,11◊,12◊,13◊,14◊,15◊,16◊,17◊,18◊,19◊,20◊,21◊,22◊
Juglandaceae					
*Juglans regia* L./UZ-53/T	Akhrot	Ft Lf / Bk	rfo, int rfo, ext	Brain and physical weakness **Toothache**	1©, 2©, 3▲, 4▲, 5◊, 6◊, 7◊, 8◊,9◊,10▲,11◊,12◊,13◊,14◊,15◊,16◊,17◊,18▲,19▲,20▲,21◊,22▲
Lamiaceae					
*Ajuga bracteosa* Wall. ex Benth./UZ-94/H	Rattibotti	Ae Lf	exr, int exr, int	**Blood purifier,** Pimples Inflammation, Earache, pain	1◊, 2◊, 3©, 4©, 5, 6▲, 7◊, 8◊,9◊,10◊,11◊,12◊,13©,14◊,15◊,16◊,17▲,18◊,19◊,20▲,21◊,22◊
*Callicarpa macrophylla* Vahl/UZ-77/S	Bengli	Lf Rt Bk	dec, ext inf, int pas, ext	**Alleviating pain in rheumatism**, Diarrhea, Dysentery Relieving rashes of tongue Wounds and cuts	1◊, 2◊, 3◊, 4◊, 5◊, 6◊, 7◊, 8◊,9◊,10◊,11◊,12◊,13◊,14◊,15◊,16◊,17◊,18◊,19◊,20◊,21◊,22◊
*Mentha spicata* L./UZ-132/H	Bebrii	Lf	pas, int	**Cooling agent, Gastric problem**	1◊, 2◊, 3◊, 4◊, 5◊, 6◊, 7◊, 8◊,9◊,10◊,11, ▲,12◊,13◊,14◊,15◊,16◊,17◊,18◊,19©,20◊,21◊,22◊
*Mentha longifolia* (L.) L./UZ-83/H	Podina	Wp Lf	dec, int pas, Int	Stimulant, Cough, Flatulence, **Digestive disorders**	1◊, 2◊, 3◊, 4◊, 5◊, 6◊, 7◊, 8◊,9◊,10◊,11◊,12◊,13◊,14◊,15◊,16◊,17◊,18◊,19◊,20◊,21◊,22◊
*Micromeria biflora* (Buch.-Ham. ex D.Don) Benth/UZ-76/S	Shahibooti	Lf Lf	oil, int jui, int	Headache **Digestive disorders**	1◊, 2▲, 3◊, 4◊, 5©, 6▲, 7◊, 8◊,9◊,10◊,11◊,12◊,13◊,14◊,15◊,16◊,17◊,18◊,19◊,20◊,21◊,22
*Ocimum sanctum* L.*/*UZ-134/H	Bebrii	Lf	pas, int	**Cooling agent, Gastric problem**	1◊, 2◊, 3◊, 4◊, 5◊, 6◊, 7◊, 8◊,9◊,10◊,11, ▲,12◊,13◊,14◊,15◊,16◊,17◊,18◊,19©,20◊,21◊,22◊
*Origanum vulgare* L./UZ-102/H	Sahthar	Wp	pow, int	**Stomach-ache,** Skin infections	1◊, 2▲, 3◊, 4◊, 5◊, 6▲, 7◊, 8◊,9◊,10▲,11◊,12▲,13◊,14◊,15◊,16◊,17◊,18◊,19◊,20◊,21◊,22◊
*Otostegia limbata* (Benth.) Boiss./UZ-23/S	Chittipataki	Rt Rt	ash, ext dec, ext	**Wound healing** Skin diseases	1◊, 2◊, 3◊, 4◊, 5◊, 6©, 7◊, 8◊,9◊,10◊,11◊,12◊,13◊,14◊,15◊ ,16◊,17◊,18▲,19◊,20◊,21◊,22◊
*Phlomis bracteosa* Royle ex Benth./UZ-144/H	Cropo	Lf Rt	exr, int pou, int	Fever, **Cough** Skin diseases	1◊,2 ◊, 3◊, 4◊, 5◊, 6◊, 7◊, 8◊,9◊,10◊,11◊,12◊,13◊,14◊,15◊,16◊,17◊,18◊,19◊,20◊,21◊,22◊
*Prunella vulgaris* L./UZ-114/H	Harswa	Wp	exr, int	Relieve respiratory difficulties, Joint problems, **Gastric spasm**	1◊, 2▲, 3▲, 4▲, 5©, 6◊, 7◊, 8◊,9◊,10◊,11◊,12▲,13◊,14▲,15◊,16◊,17◊,18◊,19◊,20◊,21◊,22◊
*Salvia lanata* Salisb./UZ-127/H	Kianar	Ae Rt	vef, int pow, int	Cough, **Common cold** Ease bowel evacuation	1◊, 2▲, 3▲, 4▲, 5◊, 6◊, 7◊, 8◊,9◊,10◊,11◊,12◊,13◊,14◊,15◊,16◊,17◊,18◊,19◊,20◊,21◊,22◊
*Thymus linearis* Benth. UZ-64/H	Chikal	Wp Ae	jui, int pow, int	Stomachache, Liver complaints **Cough**	1◊, 2◊, 3▲, 4▲, 5◊, 6◊, 7◊, 8◊,9◊,10▲,11◊,12▲,13©,14▲,15▲,16◊,17◊,18◊,19◊,20◊,21◊,22▲
Lythraceae					
*Punica granatum* L./UZ-05/S	Darhou	Sd Pl Fr Fr	pow, int jui, int rfo, int exr, int	Stomachache Cardiac problems, **Dysentery**, Diarrhea	1◊, 2◊, 3▲, 4▲, 5◊, 6▲, 7◊, 8◊,9◊,10◊,11◊,12◊,13◊,14◊,15◊,16▲,17▲,18▲,19▲,20◊,21◊,22▲
Malvaceae					
*Abutilon ramosum* (Cav.) Guill. & Perr./UZ-35/S	Shrub	Rt	pow, int	**Stomach ailment**	1◊, 2◊, 3◊, 4◊, 5◊, 6◊, 7◊, 8◊,9◊,10◊,11◊,12◊,13◊,14◊,15◊,16◊,17◊,18◊,19◊,20◊,21◊,22◊
*Malva parviflora* L./UZ-45/H	Sonchal	Lf	dec, int	**Constipation**	1◊, 2◊, 3◊, 4◊, 5◊, 6©, 7◊, 8◊,9◊,10◊,11◊,12◊,13◊,14◊,15◊,16◊,17◊,18◊,19◊,20◊,21©,22◊
*Malvastrum coromandelianum* (L.) Garcke/UZ-56/H	Bariar	Ae	dec, int	**Kill worms**, Dysentery	1◊, 2◊, 3©, 4©, 5◊, 6▲, 7◊, 8◊,9◊,10◊,11◊,12◊,13◊,14◊,15◊,16◊,17◊,18◊,19◊,20◊,21◊,22◊
Meliaceae					
*Melia azadirachta* L./UZ-73/T	Daraik	Fr/Sd Lf	pow, int dec, int	Diabetes, Blood pressure, **Blood purifier** Throat infection, Jaundice, Skin problems, High fever	1◊, 2◊, 3◊, 4◊, 5◊, 6◊, 7◊, 8◊,9◊,10◊,11▲,12◊,13◊,14◊,15◊,16◊,17©,18▲,19◊,20◊,21▲,22▲
Moraceae					
*Ficus carica* L./UZ-135/T	Injeer / Barh Phugwarah	Fr Lt	rfo, int pas, ext	Constipation Wound healing, Extract thrones from skin, **Antidote**	1◊, 2©, 3©, 4©, 5◊, 6◊, 7◊, 8◊,9◊,10▲,11◊,12◊,13◊,14◊,15◊,16▲,17▲,18◊,19▲,20◊,21◊,22▲
*Ficus palmata* Forssk./UZ-126/T	Phugwarah	Fr Lt Lf Lf	rfo, int rub, ext pas, ext veg, int	Digestive problems Extract thorns from skin Skin problems **Diabetes**	1◊, 2◊, 3▲, 4, 5, 6◊, 7◊, 8◊,9◊,10◊,11◊,12◊,13©,14◊,15◊,16◊,17◊,18©,19◊,20◊,21◊,22◊
*Morus alba* L./UZ-96/T	Toot	Lf Fr	dec, int pow, int	**Throat inflammation** Cough, Cold, Constipation	1◊, 2▲, 3◊, 4◊, 5◊, 6◊, 7◊, 8◊,9◊,10◊,11◊,12◊,13©,14◊,15◊,16◊,17◊,18◊,19©,20▲,21▲,22©
*Morus nigra* L./UZ-116/T	Shatoot	Fr	rfo, int	Cough, **Constipation**	1◊, 2▲, 3◊, 4◊, 5◊, 6◊, 7◊, 8◊,9◊,10◊,11◊,12◊,13◊,14◊,15◊,16◊,17◊,18▲,19◊,20◊,21▲,22©
Myrtaceae					
*Eucalyptus camaldulensis* Schlecht/UZ-143/T	Safeda	Bk	rub, ext	**Toothache**	1◊, 2◊, 3◊, 4◊, 5◊, 6◊, 7◊, 8◊,9◊,10◊,11◊,12◊,13◊,14◊,15◊,16◊,17◊,18◊,19◊,20◊,21◊,22◊
Oleaceae					
*Jasminum officinale* L./UZ-15/S	Chambili	Rt Lf Lf	dec, int che, int pow, ext	Ringworm **Mouth ulcer** Antidandruff, Muscular pain	1◊, 2▲, 3◊, 4◊, 5◊, 6◊, 7◊, 8◊,9◊,10◊,11◊,12◊,13▲,14◊,15◊,16◊,17◊,18◊,19◊,20◊,21▲,22◊
*Olea ferruginea* Wall. ex Aitch./UZ-24/T	Kao	Lf Ol Br	dec, int exr, int rfo, ext	Scanty urination, Throat infection Anti-rheumatic **Toothache, Mouth infection**	1◊, 2©, 3◊, 4◊, 5◊, 6▲, 7◊, 8◊,9◊,10◊,11◊,12◊,13◊,14◊,15◊,16◊,17©,18©,19◊,20▲,21◊,22▲
Onagraceae					
*Oenothera rosea* L. Her. ex Aiton/UZ-06/H	Buti	Lf	inf , int	**Hepatic pain**, Kidney disorders	1◊, 2▲, 3©, 4©, 5◊, 6▲, 7◊, 8◊,9◊,10◊,11◊,12◊,13◊,14◊,15◊,16◊,17◊,18◊,19◊,20◊,21◊,22◊
Oxalidaceae					
*Oxalis corniculata* L./UZ-36/H	Khattiboti	Wp Lf Lf	exr, int pou, int dec, int	Diarrhea, Skin diseases, Dysentery, **Blood purification** Inflammation Cooling property in fever	1◊, 2▲, 3◊, 4◊, 5, 6▲, 7◊, 8©,9▲,10▲,11◊,12▲,13◊,14◊,15◊,16◊,17◊,18▲,19◊,20◊,21©,22▲
Pinaceae					
*Pinus roxburghii* Sarg./UZ-16/T	Chir	Rs	pas, ext	Wound healing, **Healing cracks in feet,** Antidote	1, 2▲, 3▲, 4▲, 5, 6, 7, 8, 9◊,10◊,11◊,12◊,13◊,14◊,15◊,16◊,17◊,18▲,19◊,20◊,21◊,22◊
Plantaginaceae					
*Plantago lanceolata* L./UZ-07/H	Ispgol	Lf Sd	dec, ext pow, int	Wound inflammation **Throat sores** Constipation	1▲, 2▲, 3©, 4©, 5◊, 6◊, 7◊, 8◊,9◊,10▲,11◊,12▲,13◊,14▲,15◊,16◊,17◊,18◊,19©,20◊,21◊,22▲
*Veronica laxa* Benth./UZ-27/H	Sriri	Wp	tea, int	Nervous system disorder, Respiratory tract, **Cardiovascular system**	1◊, 2▲, 3◊, 4◊, 5◊, 6◊, 7◊, 8◊,9◊,10◊,11◊,12◊,13◊,14◊,15◊,16◊,17◊,18◊,19◊,20◊,21◊,22◊
Poaceae					
*Chrysopogon serrulatus* Trin./UZ-86/G	Bari Gaas	Wp	pas, ext	**Used for skin care**	1◊, 2▲ , 3◊, 4◊, 5◊, 6◊, 7◊, 8◊,9◊,10◊,11◊,12◊,13◊,14◊,15◊,16◊,17◊,18◊,19◊,20◊,21◊,22◊
*Cynodon dactylon* (L.) Pers./GUZ-138/G	Khabbal	Lf	pas, ext	**Muscle and joint fractures**	1◊, 2▲, 3◊, 4◊, 5◊, 6©, 7◊, 8▲,9▲,10◊,11▲,12◊,13◊,14◊,15◊,16◊,17◊,18▲,19◊,20◊,21▲,22◊
*Dactylis glomerata* L./UZ-17/G	Gadu	Wp Wp	pas, int exr, int	Allergies Anti-tumor, **Kidney ailments,** Bladder ailments	1◊, 2▲, 3◊, 4◊, 5◊, 6◊, 7◊, 8◊,9◊,10◊,11◊,12◊,13◊,14◊,15◊,16◊,17◊,18◊,19◊,20◊,21◊,22◊
*Dichanthium annulatum* (Forssk.) Stapf/UZ-75/G	Murgah Ghass	Bk	inf, int	**Cough**	1◊, 2▲, 3◊, 4◊, 5◊, 6◊, 7◊, 8◊,9◊,10◊,11◊,12◊,13◊,14◊,15◊,16◊,17◊,18◊,19◊,20◊,21▲,22◊
*Digitalis ciliata* Trautv./UZ-124/G	Diljit	Sd	pow, int	Cardiac treatments, Anti-proliferative, **Used for suppressing tumors**	1◊, 2◊, 3◊, 4◊, 5◊, 6◊, 7◊, 8◊,9◊,10◊,11◊,12◊,13◊,14◊,15◊,16◊,17◊,18◊,19◊,20◊,21◊,22◊
*Echinochloa colona* (L.) Link/UZ-10/G	Sanawakri	Wp	inf, int	**Hemorrhage problems**	1◊, 2◊, 3◊, 4◊, 5◊, 6◊, 7◊, 8◊,9◊,10◊,11◊,12◊,13◊,14◊,15◊,16◊,17◊,18◊,19◊,20◊,21◊,22◊
*Eleusine indica* (L.) Gaertn./UZ-107/G	Madhana ghass	Rt Lf Wp	pou, int dec, int exr, int	Gonorrhea Scanty urination **Fever**, Anti-inflammatory, Jaundice	1◊, 2◊, 3◊, 4◊, 5◊, 6◊, 7◊, 8◊,9◊,10◊,11◊,12◊,13◊,14◊,15◊,16◊,17◊,18◊,19◊,20◊,21▲,22◊
*Heteropogon contortus* (L.) P. Beauv. ex Roem. & Schult./UZ-26/G	Sariyalaghass	Rt	inf, int	**Scanty urination**	1◊, 2◊, 3◊, 4◊, 5◊, 6◊, 7◊, 8◊,9◊,10◊,11◊,12◊,13◊,14◊,15◊,16◊,17◊,18◊,19◊,20◊,21◊,22◊
*Imperata cylindrica* (L.) Raeusch./UZ-115/G	Dibb	Wp	exr , int	**Asthma**, Bruises, Paralysis, Anti-inflammatory	1◊, 2▲, 3◊, 4◊, 5◊, 6◊, 7◊, 8◊,9◊,10◊,11◊,12◊,13◊,14◊,15◊,16◊,17◊,18◊,19◊,20◊,21▲,22◊
*Lolium temulentum* L./UZ-65/G	Grass	Sd	pow, int	Sedative	1◊, 2◊, 3◊, 4◊, 5◊, 6◊, 7◊, 8◊,9◊,10◊,11◊,12◊,13◊,14◊,15◊,16◊,17◊,18◊,19◊,20◊,21◊,22◊
*Setaria pumila* (Poir.) Roem. & Schult./UZ-97/G	Kangni, Loomar Gaas	Lf Lf Gr	exr, int pou, ext jui, int	Eye drops **Fast healing** Cooling agent	1◊, 2◊, 3◊, 4◊, 5◊, 6◊, 7◊, 8◊,9◊,10◊,11◊,12◊,13◊,14◊,15◊,16◊,17◊,18◊,19◊,20◊,21◊,22◊
*Themeda anathera* (Nees ex Steud.) Hack./UZ-98/G	Bari ghass	Ae	pou, ext	**Backache,** Blood purifier	1◊, 2▲, 3©, 4©, 5◊, 6◊, 7◊, 8◊,9◊,10◊,11◊,12◊,13◊,14◊,15◊,16◊,17◊,18◊,19◊,20◊,21◊,22◊
Polygalaceae					
*Polygala abyssinica* R. Br. ex Fresen./UZ-37/H	Arna	Rt	jui, ext	Evil eye, **Antidote to snake bite**	1◊, 2▲, 3◊, 4◊, 5◊, 6◊, 7◊, 8◊,9◊,10◊,11◊,12◊,13◊,14◊,15◊,16◊,17◊,18◊,19◊,20◊,21◊,22◊
Polygonaceae					
*Persicaria maculosa* Gray/UZ-51/H	Ochi	Sd Rt Lf	pow, int pas, int pas, ext	Dysentery, **Cholera** Scabies Wound healing	1◊, 2◊, 3◊, 4◊, 5◊, 6◊, 7◊, 8◊,9◊,10◊,11◊,12◊,13◊,14◊,15◊,16◊,17◊,18◊,19◊,20◊,21◊,22◊
*Polygonum ramosissimum* Michx./UZ-40/H	Bannali	Wp	pas, int	**Urinary tract infection**	1◊, 2◊, 3◊, 4◊, 5◊, 6◊, 7◊, 8◊,9◊,10◊,11◊,12◊,13◊,14◊,15◊,16◊,17◊,18◊,19◊,20◊,21◊,22◊
*Rumex hastatus* D.Don/UZ-59/H	Jnglipalak	Lf Rt	boi, int pas, ext	Constipation **Skin disorder**	1◊, 2▲, 3▲, 4▲, 5◊, 6◊, 7◊, 8◊,9◊,10◊,11◊,12◊,13◊,14◊,15◊,16◊,17◊,18▲,19◊,20◊,21◊,22◊
Primulaceae					
*Anagallis arvensis* L./UZ-47/H	Billibooti	Wp Wp	exr, int pas, ext	**Lowering fever**, Depression, Tuberculosis, Liver problems, Epilepsy Improving the complexion, especially for freckle	1◊, 2◊, 3◊, 4◊, 5◊, 6◊, 7◊, 8◊,9◊,10◊,11◊,12◊,13◊,14◊,15◊,16◊,17◊,18◊,19◊,20◊,21◊,22◊
*Androsace rotundifolia* Hardw./UZ-58/H	Thandijarri	Rh Lf	ext, int inf, int	Cataract **Stomachache**, Emetic	1◊, 2▲, 3©, 4©, 5◊, 6◊, 7◊, 8◊,9◊,10◊,11◊,12▲,13◊,14◊,15◊,16◊,17◊,18◊,19◊,20◊,21◊,22◊
Pteridaceae					
*Adiantum tenerum* Sw./UZ-130/F	Hansraj	Fd	jui, int pas, ext	Cough, Fever, Dysentery, **Ulcers** Burning sensation, Epileptic fits	
*Onychium japonicum* (Thunb.) Kunze/UZ-28/F	Pathba	Lf & Rh	jui, int	Dysentery, **Diarrhea**	
*Pteris vittata* L./UZ-149/F	Nanore	Fd	pas, ext	**Wound healing**	1◊, 2◊, 3◊, 4◊, 5◊, 6◊, 7◊, 8◊,9◊,10◊,11◊,12◊,13◊,14◊,15◊,16◊,17◊,18◊,19◊,20◊,21◊,22◊
Ranunculaceae					
*Clematis grata* Wall./UZ-89/S	Tootal	Lf In	dec, int inf, int	Diabetes **Cough**	1◊, 2▲, 3©, 4©, 5◊, 6▲, 7◊, 8◊,9◊,10◊,11◊,12◊,13◊,14◊,15◊,16◊,17◊,18◊,19◊,20◊,21◊,22◊
*Ranunculus arvensis* L*./*UZ-80/H	Chachumba	Ae	coo, int	**Asthma**	1◊, 2◊, 3◊, 4◊, 5◊, 6◊, 7◊, 8◊,9◊,10◊,11◊,12◊,13◊,14◊,15◊,16◊,17◊,18▲,19◊,20◊,21◊,22◊
*Ranunculus muricatus* L./UZ-91/H	Kor-Kandoli	Ae	mix, int	**Asthma**	1◊, 2◊, 3©, 4©, 5◊, 6©, 7◊, 8◊,9◊,10◊,11◊,12◊,13◊,14◊,15◊,16◊,17◊,18◊,19◊,20◊,21◊,22▲
*Thalictrum revolutum* DC./UZ-112/H	Beni	Wp	jui, int	Blood purifier, **Curing fever**	1◊, 2◊, 3◊, 4◊, 5◊, 6◊, 7◊, 8◊,9◊,10◊,11◊,12◊,13◊,14◊,15◊,16◊,17◊,18◊,19◊,20◊,21◊,22◊
Rosaceae					
*Cotoneaster racemiflora* Wall. ex Lindl./UZ-08/S	Luni	Lf	tea, int	**Stop bleeding and pus**	1◊, 2◊, 3◊, 4◊, 5◊, 6◊, 7◊, 8◊,9◊,10◊,11◊,12◊,13◊,14◊,15◊,16◊,17◊,18◊,19◊,20◊,21◊,22◊
*Duchesnea indica* (Jacks.) Focke/UZ-48/H	Budimewa	Fl Wp	boi, int exr, int	Blood circulation Swelling, Boils, **Burns**	1◊, 2▲, 3▲, 4▲, 5◊, 6▲, 7◊, 8◊,9◊,10◊,11▲,12◊,13◊,14◊,15◊,16◊,17◊,18◊,19▲,20◊,21◊,22◊
*Eriobotrya japonica* (Thunb.) Lindl./UZ-29/T	Loukat	Wp Lf Fr Fl	dec, int pas, int rfo, int boi, int	Cough, Constipation Nose bleeds, Coughing up blood, Diarrhea, **Depression**, Skin diseases, Digestive disorders, Respiratory problems Common cold	1◊, 2◊, 3▲, 4▲, 5◊, 6◊, 7◊, 8◊,9◊,10◊,11◊,12◊,13◊,14◊,15◊,16◊,17◊,18◊,19◊,20◊,21◊,22◊
*Fragaria nubicola* (Lindl. ex Hook.f.) Lacaita/UZ-148/H	Boodimava	Fr	jui , int	Diarrhea, **Dysentery,** Diabetes, Sexual diseases	1◊, 2▲, 3▲, 4▲, 5◊, 6◊, 7▲, 8◊,9◊,10◊,11◊,12◊,13◊,14◊,15◊,16◊,17◊,18◊,19◊,20◊,21◊,22▲
*Potentilla reptans* L./UZ-137/H	Gul bota	Wp	jui, int	**Diarrhea,** Intestinal infections	1◊, 2◊, 3◊, 4◊, 5◊, 6◊, 7◊, 8◊,9◊,10◊,11◊,12◊,13◊,14◊,15◊,16◊,17◊,18◊,19◊,20◊,21◊,22◊
*Prunus armeniaca* L./UZ-70/T	Hari, Khubani, Apricot	Fr Sd	eat , int oil, ext	**Constipation** Softening effect on the skin	1◊, 2◊, 3©, 4©, 5◊, 6◊, 7◊, 8◊,9◊,10◊,11◊,12◊,13◊,14◊,15◊,16◊,17◊,18◊,19◊,20◊,21◊,22◊
*Prunus domestica* L./UZ-87/T	Alucha	Fr	eat, int	Irregular menstruation, **Miscarriage**, Alcoholic beverages and liqueur	1◊, 2◊, 3©, 4©, 5◊, 6◊, 7◊, 8◊,9◊,10◊,11◊,12◊,13◊,14◊,15◊,16◊,17◊,18◊,19◊,20◊,21◊,22◊
*Prunus persica* (L.) Batsch/UZ-68/T	Aruu, Peach	Lf	jui, int	**Gastritis**, Cough, Bronchitis, Kill worms	1◊, 2◊, 3©, 4©, 5◊, 6▲, 7◊, 8◊,9◊,10◊,11◊,12◊,13◊,14◊,15◊,16◊,17◊,18▲,19◊,20◊,21◊,22◊
*Pyrus malus* L./UZ-99/T	Saib	Fr	jui, int	Rheumatism, **Hypertension**, Tonic for vigorous body, Strengthen bones, face spots	1◊, 2◊, 3©, 4©, 5◊, 6◊, 7◊, 8◊,9◊,10◊,11◊,12◊,13◊,14◊,15◊,16◊,17◊,18◊,19◊,20◊,21◊,22◊
*Pyrus pashia* Buch. -Ham. ex D. Don/UZ-38/T	Tangi	Fr Fr	pas, int eat, int	Dark eye circles **Constipation**	1◊, 2▲, 3©, 4◊, 5◊, 6◊, 7◊, 8◊,9◊,10▲,11◊,12◊,13◊,14◊,15◊,16◊,17◊,18◊,19◊,20◊,21◊,22◊
*Rosa brunonii* Lindl./UZ-121/S	Chal	Fl Fl	pow, int dec, int	Heart tonic, Skin diseases **Constipation**	1◊, 2▲, 3©, 4©, 5◊, 6©, 7◊, 8◊,9◊,10◊,11◊,12▲,13◊,14▲,15◊,16◊,17◊,18©,19◊,20◊,21◊,22◊
*Rosa indica* L./UZ-30/S	Galab	Fl	exr , int	**Eye diseases**, Stomachache, Fever, Pneumonia	1◊, 2◊, 3◊, 4◊, 5◊, 6◊, 7◊, 8◊,9◊,10◊,11◊,12◊,13◊,14◊,15◊,16◊,17◊,18▲,19◊,20◊,21◊,22◊
*Rubus ellipticus* Sm./UZ-109/S	Aakhara	Lf Rt / Bk	dec, int pow, int exr, int	Diarrhea, Bleeding Against skin diseases especially female genitalia **Dysentery**	1◊, 2▲, 3◊, 4◊, 5◊, 6◊, 7◊, 8◊,9◊,10◊,11▲,12◊,13◊,14◊,15◊,16◊,17◊,18▲,19◊,20◊,21◊,22◊ RT/ BK
*Rubus niveus* Thunb*.*/UZ-19/S	Garachi	Lf Rt	pow, int dec, int	Diarrhea, Fever, **Blood purifier** Dysentery, Colic, Pain, Whooping Cough	1◊, 2▲, 3©, 4©, 5◊, 6◊, 7◊, 8◊,9◊,10◊,11◊,12◊,13◊,14◊,15◊,16◊,17◊,18◊,19◊,20◊,21◊,22◊
*Spiraea canescens* D. Don/UZ-57/H	Jhar, Mariala	Rt Sd	exr, int pow, int	Enema and to treat venereal conditions **Insomnia**	1◊, 2▲, 3◊, 4◊, 5◊, 6◊, 7◊, 8◊,9◊,10◊,11◊,12◊,13◊,14◊,15◊,16◊,17◊,18◊,19◊,20◊,21◊,22◊
Rubiaceae					
*Galium aparine* L./UZ-119/H	Boora	Wp Wp	mix, int jui, int	Constipation, Stomachic diseases **Scanty urination,** Constipation	1◊, 2▲, 3▲, 4▲, 5◊, 6◊, 7▲, 8, 9◊,10▲,11◊,12◊,13◊,14◊,15◊,16◊,17◊,18◊,19◊,20◊,21◊,22◊
Rutaeae					
*Zanthoxylum armatum* DC./UZ-106/S	Timber	Sd / Bk Bk	rfo, int rub, int	Tonic, Cholera, fever, Dyspepsia, **Stomachache** Toothache	1◊, 2▲, 3©, 4©, 5◊, 6▲, 7◊, 8◊,9◊,10▲,11▲,12◊,13◊,14◊,15◊,16◊,17◊,18©,19◊,20◊,21◊,22◊
Salicaceae					
*Populus nigra* L./UZ-123/T	Sfeeda	Bk Bk Lf	exr, int pas, ext pas, ext	Arthritis, Gout, **Lower back pain,** Urinary complaints, Gout, Digestive disorders, Liver disorders, Fever, Relieve the pain of menstrual cramps Hemorrhoids Infected wounds and sprains, Caries of teeth and bones	1◊, 2▲, 3◊, 4◊, 5◊, 6◊, 7◊, 8◊,9◊,10◊,11◊,12◊,13◊,14◊,15◊,16◊,17◊,18◊,19◊,20◊,21◊,22◊
Sapindaceae					
*Aesculus indica* (Wall. ex Camb.) Hook./UZ-141/T	Bankhore	Fr Sd	rfo, int pow, int	Colic, **Rheumatism** Leucorrhoea	1◊, 2◊, 3©, 4©, 5◊, 6◊, 7◊, 8◊,9◊,10▲,11◊,12◊,13◊,14◊,15◊,16◊,17◊,18◊,19◊,20©,21◊,22◊
*Dodonaea viscosa* (L.) Jacq./UZ-139/S	Snathaa	Lf	pow, ext	**Healing agent**	1◊, 2◊, 3◊, 4◊, 5◊, 6◊, 7◊, 8◊,9◊,10◊,11◊,12◊,13◊,14◊,15◊,16◊,17◊,18©,19◊,20◊,21◊,22◊
Scrophulariaceae					
*Verbascum thapsus* L./UZ-18/H	Gidar	Lf Wp Rt	dec, ext inf, int ext, int	Sunburn, Ulcers, Tumors, **Piles,** Sedative, Narcotic Toothache, Relieve cramps, Convulsions	1◊, 2▲, 3▲, 4▲, 5◊, 6▲, 7◊, 8◊,9◊,10▲,11◊,12◊,13▲,14◊,15◊,16◊,17◊,18▲,19◊,20◊,21◊,22◊
Simaroubaceae					
*Ailanthus altissima* (Mill.) Swingle/UZ-92/T	Draviyae	Bk Lf	inf, int ext, int	**Dysentery**, Diarrhea Blood purifier	1◊, 2▲, 3◊, 4◊, 5◊, 6◊, 7◊, 8◊,9◊,10◊,11◊,12◊,13◊,14◊,15◊,16◊,17◊,18◊,19◊,20◊,21◊,22◊
Solanaceae					
*Solanum villosum* Mill./UZ-09/H	Kaach, Maach	Lf Fr	dec, int rfo, Int	Scanty urination **Tongue infection in children**	1◊, 2◊, 3◊, 4◊, 5◊, 6◊, 7◊, 8◊,9◊,10◊,11◊,12◊,13◊,14◊,15◊,16◊,17◊,18◊,19◊,20◊,21◊,22◊
Urticaceae					
*Debregeasia salicifolia* (D. Don) Rendle/UZ-39/S	Sindari	Ae	inf, ext	Eczema, **Dermatitis**	1◊, 2◊, 3©, 4©, 5◊, 6©, 7◊, 8◊,9◊,10◊,11◊,12◊,13◊,14◊,15◊,16◊,17◊,18©,19◊,20◊,21◊,22◊
*Urtica dioica* L./UZ-49/H	Kinjii	Rt Rt	inf, int pas, int	**Scanty urination** Anti-allergic	1◊, 2◊, 3◊, 4◊, 5◊, 6◊, 7▲, 8◊,9◊,10◊,11▲,12◊,13▲,14◊,15▲,16◊,17◊,18◊,19◊,20◊,21◊,22▲
Valerianaceae					
*Valeriana jatamansi* Jones./UZ-60/H	Mushk bala	Rh	exr, int	**Intestinal pain**, Neurosis, Constipation	1◊, 2◊, 3▲, 4▲, 5◊, 6◊, 7▲, 8◊,9◊,10▲,11◊,12▲,13◊,14◊,15◊,16◊,17◊,18◊,19◊,20◊,21◊,22▲
Verbenaceae					
Vitex *agnus-castus* L./UZ-88/T		Lf	exr, int	Female reproductive system disorders, Scanty urination, Digestive disorders, Anxiety, **Stomachache**	1◊, 2◊, 3◊, 4◊, 5◊, 6◊, 7◊, 8◊,9◊,10◊,11◊,12◊,13◊,14◊,15◊,16◊,17 **Stomachache** ◊,18◊,19◊,20◊,21◊,22◊
*Verbena officinalis* L./UZ-67/H	Chandni	Wp	inf, int	Scanty urination, **Reduces inflammation,** Control bleeding, Malaria, Nervous exhaustion, Depression, Asthma, Migraine, Jaundice	1◊, 2▲, 3◊, 4◊, 5◊, 6◊, 7◊, 8©,9◊,10◊,11◊,12◊,13◊,14◊,15◊,16◊,17◊,18◊,19◊,20◊,21◊,22◊
Violaceae					
*Viola odorata* L./UZ-100/H	Banafshan	Wp	boi, int	Flu, Cough, **Jaundice**	1◊, 2◊, 3◊, 4◊, 5▲, 6◊, 7◊, 8◊,9◊,10▲,11◊,12◊,13◊,14◊,15◊,16▲,17◊,18◊,19◊,20◊,21◊,22◊

Habit: H, Herbs; S, Shrubs; T, Trees; F, Ferns; E, Epiphyte. Part(s) Used: Lf, Leaf; Fr, Fruit; Rt, Root; St, Stem; Ae, Aerial Parts; Wp, Whole Plant; Fd, Fronds; Sd, Seed; Fl, Flower; Bk, Bark; Bl, Bulb; Rh, Rhizome; In, Inflorescence; Sh, Shoot; Lt, Latex; Br, Branches; Rs, Resin; Gr, Grain; Pd, Pods; Pl, Pulp; Ol, Oil. Method of preparation: pow, Powder; dec, Decoction; ext, Extract; pas, Paste; jui, Juice; pou, Poultice; inf, Infusion; che, Chewed; veg, Vegetable; rub, Rubbing; eat, Eaten; coo, Cooked; boi, Boiled; flu, Fluid; fra, Fragrance, mix, Mixture. Mode of Aministration: int, Internal; exr, External. Previous use reports: (©) = plants with similar use(s); (▲) = plants with dissimilar use (s); (◊) = plants not reported in previous study; bold written highlights the frequent use for a given plant. 1: Mahmood et al. [64]; 2: Khan et al. [65]; 3: Amjadet al [41].; 4: Shaheenet al [47].; 5: Ishtiaq et al. [66]; 6: Amjad et al. [67]; 7: Gilani et al. [68]; 8: Gulshan et al. [69]; 9: Mahmood et al. [70];10: Rana et al. [71]; 11: Jadhav [72];12: Gidey et al. [73]; 13: Dar [74];14: Bano et al. [39]; 15: Khan et al. [75]; 16: Hussain et al. [76] 17: Ullah and Bibi [77]; 18: Qaseem et al. [40]; 19: Aziz et al. [14]; 20. Ahmad et al. [32]; Umair et al. [78]; 22. Hussain et al. [76]

**Fig. 4:**
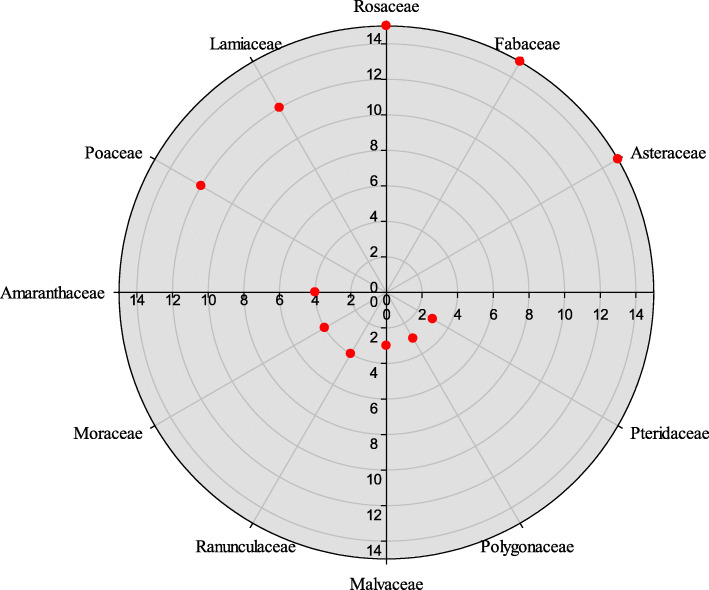
Family contribution of medicinal flora in the study area

Asteraceae, Fabaceae, and Rosaceae were the dominant families having 15 species each, followed by Lamiaceae and Poaceae having 12 species each (Fig. 4). Our results are in accordance with Amjad et al. [41], Kayani et al. [20], and Tariq et al. [21]. The prevalence of these families might be due to their abundance and easy accessibility in the study area. Moreover, majority of the reported species of these families possess significant pharmaceutical, pharmacological and organoleptic properties [82–85].

### Plant part(s) used

Herbal recipes were prepared using different plant parts in investigated area. As shown in Table 2, leaves, whole plant, and roots were the most preferred plant parts used in herbal preparations (30.2, 16.6, and 14.4%, respectively). These findings were similar as reported earlier from Pakistan and other countries [40, 44, 86–89]. Local preference of leaves in herbal recipes is because of their collection and availability. Leaves, whole plant, and roots are rich in health beneficial secondary metabolites that contribute significantly in the prevention and treatment of various health disorders [6, 44, 90–92]. Though, local inhabitants prefer to use whole plant and roots but their use is not recommended, as exploitation of whole plant species or their uprooting could cause harmful effect on regeneration and may cause species extension [93, 94].

### Method of preparation and administration

**Fig. 5:**
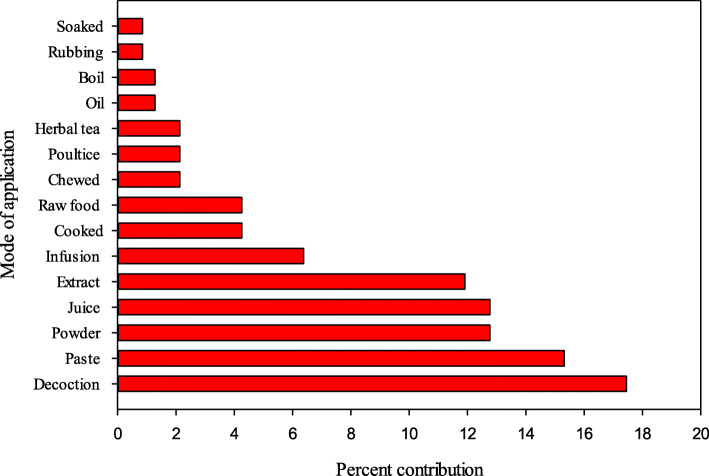
Method of preparation of herbal recipes

Different recipes were prepared from medicinal plants by using different methods based on the actual site and type of disease treated. Decoction was the most common method (41 sp.; 17%) for preparation of herbal recipes, followed by paste (36 sp.; 15%), powder (30 sp.; 13%), extract (28 sp.; 12%), and juice (30 pp.; 13%) (Fig. 5). Our findings are supported by previous documentation [17, 40, 41, 80, 95, 96]. Ease of preparation might be a reason for the extensive use of decoctions to treat aliments, as it can be prepared by mixing specific part of plants with soup, tea, water, honey, milk, and butter [97]. The availability of active metabolic compounds might increase due to the fact of heating which speed up the biological reactions [98–101]. Sometimes, whole plants were used in herbal preparations. Most of the herbal recipes were prepared using single plant species assuming non-toxicity, palatability, and high efficacy. Some recipes were based on application of two or more plants to attain maximum therapeutic effects. The amount of medicinal plants and frequency of dose are based on patient condition, health, age, and disease severity. In the study area, constipation was commonly treated using the fruits of *Ficus carica*, and for adults 4–5 fruits were used, while 2–3 were regarded as effective for children. The frequent mode of application was internal (76.2%) and only few preparations were applied topically (Table 2), as paste, body wash, or rubbed on the affected body parts. These findings were comparable to previous reports [20, 40, 44].

### Informant consensus factor

**Fig. 6:**
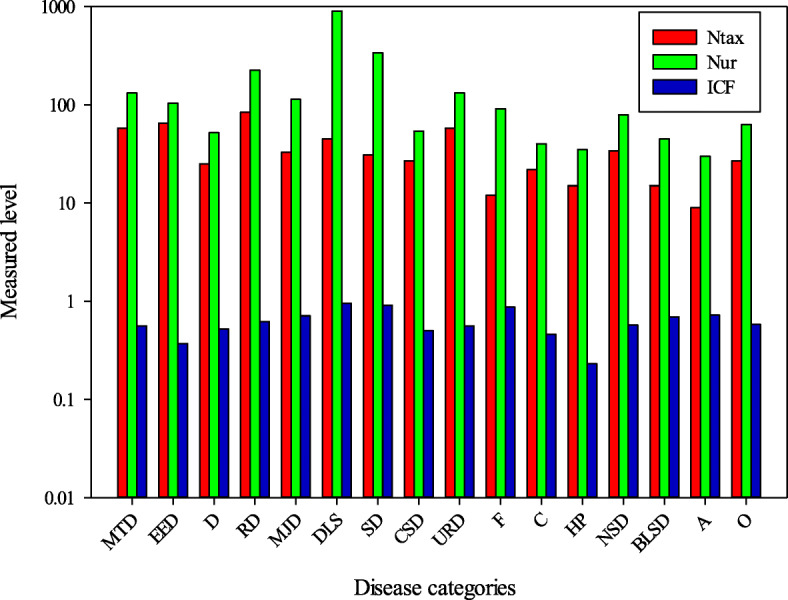
Informant consensus factor of diseases with use reports and total number of species used. *Ntax* total species used by all the informants for group of ailments, *UR* total number of use reports in each group of disease, *ICF* informant consensus factor, *MTD* mouth-throat diseases, *EED* eye and ear diseases, *D* diabetes, *RD* respiratory diseases, *MJD* muscular and joint diseases, *DLS* digestive system and liver diseases, *SD* skin diseases, *CSD* circulatory system diseases, *URD* urinary and reproductive system diseases, *F* fever, *C* cancer, *HP* hair problem, *NSD* nervous system disorder, *BLSD* blood and lymphatic system diseases, *A* antidotes, *O* others

A total 127 emic use reports were categorized in to 16 etic use categories (Table 2S). The informant consensus factor was analyzed based on disease categories, as a single plant might be used to treat 3–4 or more disease categories. A high informant consensus factor (ICF) value reflects high dependence of local inhabitants on medicinal plants [102] and low ICF values indicate less consistency of informant’s knowledge. Ailments were classified in to 18 categories to develop informant consensus. The ICF value ranged from 0.23 to 0.95. The maximum value of ICF was estimated for digestive disease category (0.95) followed by skin problems and respiratory disorders (Fig. 6). This was strongly supported by Qaseem et al. [40], Umari et al. [78], Ullah et al. [42], Amjad et al. [41], Ahmad et al. [44], and Bib et al. [17] who also reported the maximum ICF for digestive diseases in their investigated area. ICF values are generally influenced by the number of informants and are more significant when calculated for uses cited by many informants. In general, ICF values were high in our study, revealing that the informants tend to agree on which plants used in the treatment of common illnesses. According to Heinrich [103], high ICF can help in identifying potentially effective medicinal plants. It was observed that in our study, the highest agreement level was recorded for diseases reported as the most widespread in rural communities of the Bagh district and other areas of Pakistan. The digestive disorder was also reported as first use class by other ethnic communities across world [102, 104–109]. The prevalence of digestive disorders among the local inhabitant might be due to inadequate availability of hygienic food and drinking water and also the common inhalation of fuel wood’s smoke [40, 42, 110, 111]. Moreover, the devastating earthquake of 2005 caused extensive damage to water resources (freshwater springs) and water supply schemes, causing the drinking water quality to be very poor, with local communities usually using contaminated water. The second highest ICF was recorded for respiratory disorders which may be due to prevalence of cold and moist conditions at high altitude Kayani et al. [20]. Besides, study area is a rich source of flowering plants and mushrooms and the prevalence of pollen and spore present in air also cause respiratory problem. Skin disorders also have high ICF value, and UV radiations, unhygienic conditions, and combine family systems (where many members live together in one room or home even some time with domesticated animals) could be the possible reasons of the prevalence of skin infections in the study area.

### Relative frequency of citation and use value

Relative frequency of citation (RFC) is used to identify the highly important species in various ailments as cited by local people [31]. The value of RFC ranged between 0.93 and 0.04. *Berberis lycium* had highest RFC value (0.81). Other plant species with significant RFC value were *Ajuga bracteosa*, *Prunella vulgaris*, *Adiantum capillus-veneris*, *Desmodium polycarpum*, *Pinus roxburgii*, *Rosa brunonii*, *Punica granatum*, *Zanthoxylum armatum*, and *Jasminum mesnyi* (Table 3). The plants species with high RFC value were abundant in the area therefore the local people were much familiar with them particularly with reference to ethnomedicinal perspective over a long time period. Likewise, the plants with special properties to cure particular disease were well known among the local culture; therefore, their precise properties to treat particular disease have got famous and deep rooted. The plant species with high RFC values would be interesting for phytochemical and pharmacological profiling and possible future drug discovery, as well as authentication at a commercial level [20, 40, 44, 112].

**Table 3:** Quantitative analysis of ethnobotanical data

Scientific name	FC	RFC	Ui	UV	RelPH	RelBS	RI
*Abutilon ramosum*	3	0.04	3	0.04	0.08	0.13	10.5
*Acacia modesta*	30	0.4	33	0.44	0.17	0.25	21
*Acacia nilotica*	64	0.86	65	0.87	0.42	0.38	40
*Achillea millefolium*	38	0.15	46	0.62	0.5	0.75	62.5
*Achyranthes aspera*	50	0.67	66	0.88	0.25	0.38	31.5
*Adiantum tenerum*	19	0.25	21	0.28	0.58	0.63	60.5
*Aesculus indica*	34	0.45	39	0.52	0.33	0.5	41.5
*Ajuga bracteosa*	42	0.56	54	0.72	0.42	0.5	46
*Ailanthus altissima*	60	0.81	64	0.86	0.25	0.25	25
*Allium griffithianum*	42	0.56	48	0.64	0.25	0.13	19
*Alternanthera pungens*	10	0.13	23	0.31	0.17	0.13	15
*Amaranthus spinosus*	36	0.48	39	0.52	0.25	0.25	25
*Amaranthus viridis*	51	0.68	57	0.77	0.42	0.5	46
*Anagallis arvensis*	22	0.29	29	0.39	0.5	0.5	50
*Anaphalis adnata*	14	0.18	18	0.24	0.17	0.25	21
*Androsace rotundifolia*	6	0.08	11	0.14	0.25	0.38	31.5
*Angelica glauca*	20	0.27	27	0.36	0.17	0.25	21
*Artemisia vulgaris*	52	0.7	55	0.74	0.17	0.25	12.67
*Asplenium dalhousiae*	34	0.45	34	0.45	0.08	0.13	10.5
*Astragalus Canadensis*	8	0.1	8	0.1	0.08	0.13	0.5
*Bauhinia variegate*	44	0.59	47	0.63	0.25	0.25	50
*Berberis lycium*	60	0.81	67	0.9	0.25	0.38	31.5
*Bidens biternata*	53	0.71	61	0.82	0.17	0.25	12.65
*Callicarpa mycrophylla*	39	0.52	43	0.58	0.42	0.5	46
*Cannabis sativa*	58	0.78	60	0.81	0.33	0.5	41.5
*Capsella bursa-pastoris*	25	0.33	44	0.59	0.33	0.38	35.5
*Carissa opaca*	32	0.43	38	0.51	0.33	0.38	35.5
*Cirsium vulgare*	32	0.43	41	0.55	0.25	0.38	31.5
*Chenopodium album*	19	0.25	19	0.25	0.08	0.13	10.5
*Chrysopogon serrulatus*	4	0.05	4	0.05	0.08	0.13	10.5
*Clematis grata*	2	0.027	2	0.027	0.17	0.25	21
*Commelina benghalensis*	5	0.06	5	0.08	0.13		10.5
*Convolvulus arvensis*	52	0.7	58	0.78	0.58	0.63	60.5
*Conyza Canadensis*	40	0.54	47	0.63	0.58	0.5	54
*Cornus macrophylla*	8	0.1	12	0.16	0.08	0.13	10.5
*Cotoneaster racemiflora*	2	0.02	2	0.02	0.17	0.25	21
*Crepis multicaulis*	10	0.13	10	0.13	0.08	0.13	10.5
*Crotalaria juncea*	2	0.02	4	0.05	0.33	0.5	41.5
*Cuscuta reflexa*	57	0.77	60	0.81	0.17	0.25	21
*Cynodon dactylon*	60	0.81	64	0.86	0.08	0.13	10.5
*Cynoglossum lanceolatum*	69	0.93	70	0.94	0.08	0.13	10.5
*Cyperus rotundus*	4	0.05	9	0.12	0.25	0.13	19
*Dactylis glomerata*	6	0.08	19	0.25	0.42	0.63	52.5
*Dalbergia sissoo*	60	0.81	62	0.83	0.5	0.63	56.5
*Debregeasia salicifolia*	33	0.44	38	0.51	0.17	0.25	21
*Desmodium elegans*	26	0.35	26	0.35	0.17	0.25	21
*Dichanthium annulalum*	10	0.13	10	0.13	0.17	0.25	21
*Dicliptera bupleuroides*	10	0.13	20	0.27	0.25	0.38	31.5
*Digitalis ciliata*	8	0.1	16	0.21	0.25	0.38	31.5
*Dodonaea viscosa*	60	0.81	60	0.81	0.25	0.38	31
*Dryopteris filix-mas*	60	0.81	60	0.81	0.08	0.13	10.5
*Duchesnea indica*	30	0.4	38	0.51	0.17	0.25	21
*Echinochloa colona*	2	0.02	4	0.05	0.17	0.25	21
*Elaeagnus umbellate*	42	0.56	54	0.72	0.33	0.38	35.5
*Eleusine indica*	9	0.12	14	0.18	0.42	0.63	52.5
*Eriobotrya japonica*	61	0.82	63	0.85	0.33	0.5	41.5
*Eucalyptus camaldulensis*	43	0.58	46	0.62	0.08	0.13	6.33
*Euphorbia helioscopia*	64	0.86	68	0.91	0.25	0.25	25
*Ficus carica*	61	0.82	63	0.85	0.33	0.38	19.08
*Ficus palmate*	50	0.67	70	0.94	0.42	0.5	25.42
*Fragaria nubicola*	46	0.62	48	0.64	1.00	0.63	81.5
*Galium aparine*	54	0.72	56	0.75	0.92	1.00	96.0
*Geranium rotundifolium*	46	0.62	49	0.66	0.25	0.38	31.5
*Gerbera gossypina*	38	0.51	40	0.54	0.42	0.38	40.0
*Hedera nepalensis*	20	0.27	35	0.47	0.25	0.38	31.5
*Helianthus annuus*	50	0.67	61	0.82	0.58	0.63	60.5
*Heteropogon contortus*	10	0.13	14	0.18	0.17	0.25	21.0
*Impatiens edgeworthii*	14	0.18	25	0.33	0.25	0.38	31.5
*Imperata cyilindrica*	7	0.09	24	0.32	0.42	0.5	46
*Indigofera linifolia*	24	0.32	28	0.37	0.08	0.13	10.5
*Ipomoea purpurea*	49	0.66	52	0.7	0.33	0.5	41.5
*Jasminum officinale*	54	0.72	59	0.79	0.33	0.5	25.33
*Juglans regia*	58	0.78	66	0.89	0.17	0.25	21
*Justicia adhatoda*	40	0.54	53	0.71	0.33	0.25	29
*Lathyrus aphaca*	15	0.2	17	0.22	0.08	0.13	0.5
*Launaea procumbens*	15	0.2	27	0.36	0.17	0.25	21
*Lespedeza juncea*	5	0.06	8	0.1	0.17	0.13	15
*Lolium temulentum*	2	0.02	3	0.04	0.08	0.13	10.5
*Lotus corniculatus*	5	0.06	7	0.09	0.5	0.75	62.7
*Malva parviflora*	65	0.87	65	0.87	0.08	0.13	0.5
*Malvastrum coromandelianum*	42	0.56	48	0.64	0.17	0.13	5
*Maytenus nemorosa*	4	0.05	7	0.09	0.17	0.25	21
*Medicago polymorpha*	44	0.59	49	0.66	0.17	0.13	15
*Melia azadrachta*	65	0.87	69	0.93	0.58	0.88	73
*Mentha spicata*	55	0.74	57	0.77	0.17	0.25	21
*Mentha longifolia*	64	0.86	78	1.05	0.5	0.38	44
*Micromeria biflora*	33	0.44	53	0.71	0.17	0.13	15
*Mimosa pudica*	3	0.04	5	0.06	0.83	0.1	91.5
*Morus alba*	61	0.82	66	0.89	0.33	0.38	35.5
*Morus nigra*	44	0.59	60	0.81	0.25	0.25	50
*Nerium oleander*	50	0.67	54	0.72	0.5	0.63	56.5
*Ocimum sanctum*	55	0.74	57	0.77	0.17	0.25	21
*Oenothera rosea*	15	0.2	20	0.27	0.17	0.25	35.5
*Olea ferruginea.*	64	0.86	76	1.02	0.58	0.75	38.08
*Onychium japonicum*	17	0.22	24	0.32	0.17	0.13	15
*Origanum vulgare*	16	0.21	28	0.37	0.25	0.25	50
*Otostegia limbata*	3	0.04	8	0.1	0.17	0.13	15
*Oxalis corniculata*	60	0.81	65	0.87	0.5	0.5	50
*Persicaria maculosa*	7	0.09	15	0.2	0.42	0.38	40
*Phlomis bracteosa*	8	0.1	11	0.14	0.25	0.25	50
*Pinus roxburghii*	60	0.81	64	0.86	0.25	0.25	50
*Plantago lanceolata*	30	0.4	36	0.48	0.25	0.25	50
*Polygala abyssinica*	2	0.02	2	0.02	0.08	0.13	10.5
*Polygonatum geminiflorum*	6	0.08	11	0.16	0.58	0.5	54
*Polygonum ramosissimum*	5	0.06	5	0.06	0.08	0.13	10.5
*Populus nigra*	53	0.71	56	0.75	0.08	0.13	10.5
*Potentilla reptans*	4	0.05	6	0.08	0.17	0.13	15
*Prunella vulgaris*	31	0.41	48	0.64	0.25	0.38	31.5
*Prunus armeniaca*	35	0.47	38	0.51	0.25	0.38	31.5
*Prunus domestica*	39	0.52	42	0.56	0.33	0.25	29
*Prunus persica*	43	0.58	49	0.66	0.42	0.5	46
*Pteris vittata*	21	0.28	27	0.36	0.08	0.13	10.5
*Punica granatum*	68	0.91	72	0.97	0.42	0.38	40
*Pyrus malus*	46	0.62	51	0.68	0.5	0.5	50
*Pyrus pashia*	38	0.51	38	0.51	0.33	0.25	29
*Ranunculus arvensis*	29	0.39	29	0.39	0.08	0.13	10.5
*Rauanculus muricatus*	20	0.27	20	0.27	0.08	0.13	1050
*Ricinus communis*	28	0.37	51	0.68	0.5	0.5	50
*Rosa brunoni*	50	0.67	52	0.7	0.25	0.38	31.5
*Rosa indica*	69	0.93	71	0.95	0.08	0.13	0.5
*Rubus ellipticus*	58	0.78	60	0.81	0.42	0.5	46
*Rubus niveus*	55	0.74	59	0.79	0.67	0.63	65
*Rumex hastatus*	51	0.68	55	0.74	0.17	0.25	21
*Salvia lanata*	18	0.24	28	0.37	0.25	0.25	50
*Sarcococca saligna*	2	0.02	6	0.08	0.25	0.38	31.5
*Setaria pumila*	6	0.08	13	0.17	0.25	0.38	31.5
*Silybum marianum*	25	0.33	53	0.71	0.67	0.63	65
*Solanum villosum*	66	0.89	69	0.93	0.17	0.25	21
*Sonchus oleraceus*	52	0.7	52	0.7	0.08	0.13	10.5
*Spiraea canescens*	14	0.18	17	0.22	0.17	0.25	21
*Swertia cordata*	3	0.04	9	0.12	0.33	0.25	29
*Tagetes minuta*	14	0.2	25	0.35	0.09	0.41	115
*Taraxacum officinale*	54	0.72	59	0.79	0.25	0.38	31.5
*Thalictrum revolutum*	11	0.14	16	0.21	0.17	0.25	21
*Themeda anathera*	16	0.21	18	0.24	0.17	0.25	21
*Thymus linearis*	22	0.29	39	0.52	0.25	0.25	50
*Torilis japonica*	14	0.18	19	0.25	0.5	0.63	32
*Trichodesma indicum*	14	0.18	19	0.24	0.25	0.38	31.5
*Trifolium resupinatum*	20	0.27	27	0.36	0.08	0.13	10.5
*Urtica dioica*	44	0.59	47	0.63	0.17	0.25	21
*Valeraina jatamansi*	26	0.35	30	0.4	0.25	0.25	25
*Verbascum Thapsus*	10	0.13	12	0.16	0.83	0.75	79
*Verbena officinalis*	25	0.33	27	0.36	0.75	0.88	81
*Veronica laxa*	9	0.12	13	0.17	0.83	0.75	79
*Viburnum grandiflorum*	53	0.71	57	0.77	0.17	0.25	21
*Vicia sativa*	44	0.59	51	0.68	0.83	0.88	85.5
*Vincetoxicum hirundinaria*	15	0.2	28	0.37	0.17	0.13	15
*Viola odorata*	62	0.83	65	0.87	0.25	0.25	25
Vitex *agnus-castus*	54	0.72	58	0.78	0.5 0	0.5	50
*Xanthium strumarium*	62	0.83	128	1.72	0.33	0.38	35.5
*Zanthoxylum armatum*	68	0.91	75	1.01	0.42	0.5	46

*FC* frequency of citation, *RFC* relative frequency of citation, *Ui* use reports cited by each respondent for given species, *UV* use value, *Rel*. *PH* relative number of pharmacological properties attributed to a single plant, *Rel*. *BS* relative number of body systems treated by a single species, *RI* relative importance

Use value reflects the relative importance of every species with reference to more use reports cited by local informants. The use value ranged between 1.05 and 0.08. *Mentha longifolia* (1.05), *Olea ferouginea* (1.02), and *Zanthoxylum armatum* (1.01) had high use value while other species with significantly high use value were *Solanum villosum* (0.93), *Cynoglossom lanceolatum* (0.94), *Rosa indica* (0.95), and *Punica granatum* (0.97) (Table 3)*.* UV value is directly related with use reports. Plant species with more use reports have high use value and vice versa [40, 41, 95]. These plant species are used in repetitive manner and are biologically more active [113]. It is not necessary that the plant which has low UV value become unimportant or not biologically active as the RFC and UV are constant in particular area but they may be change according to the variation in the knowledge of indigenous people from area to area or within area.

Species with high RFC and UV show high healing potential for particular disease. Species with high RFC and UV were often overharvested by inhabitants, so they are prioritized for conservation and sustainable use; otherwise, they will be extinct from the area in near future [20, 44, 114]. The ethnomedicinal knowledge is at risk because there might be no resource left for younger generations. The main reason for this is that the local inhabitants of the area, especially young generations, have little interest and understanding or knowledge about ethnomedicinal plants, and are already dependent upon allopathic medicine for their healthcare [47, 114, 115].

### Relative importance

Relative importance value is used to determine the diversity of a species for treating various ailments. *Galium aparine* (96) and *Mimosa pudica* (91) had highest RI values while *Verbena officinalis* (81.5), *Fragaria nubicola* (81), *Verbascum thapsus* (79), and *Melia azadirachta* (73) had high RI values (Table 3). It was observed that species with high RI value was used frequently for treating several ailments. The natives have too much ethnomedicinal knowledge regarding these plants. Therefore, importance of these species increase as the number of treated systems increases [41, 114, 116].

### Fidelity level

**Fig. 7:**
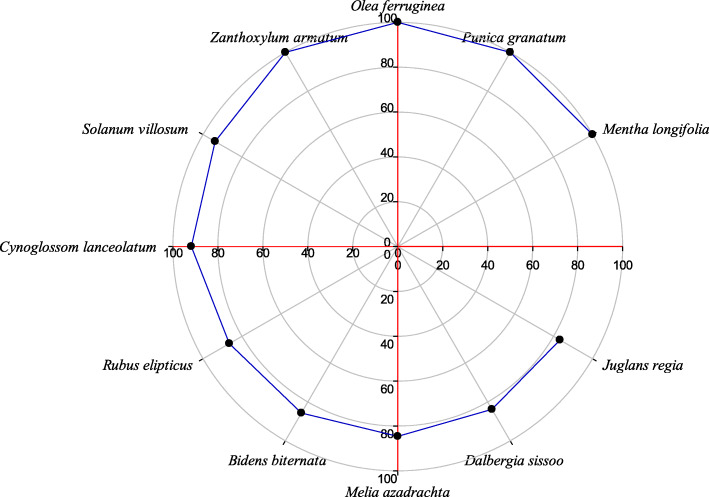
Top ranked plant species with above 80% fidelity

The fidelity value reflects the preference of particular plant species as reported by local people for curing particular ailment in the area. The FL value of reported species ranged from 18.2 to 100%. Fidelity values of four plant species viz *Mentha longifolia*, *Punica granatum*, *Zanthoxylum armatum*, and *Olea ferruginea* were found 100%, and these species were used to cure stomachache, dysentery, rheumatism, and other digestive disorders. Other medicinal plants having high FL value were *Solanum villosum* (93.8), *Cynoglossum lanceolatum* (91.8), *Dalbergia sissoo* (83.8), *Bidens biternata* (85.7%), *Rubus ellipticus* (86.5%), and *Melia azadirachta* (84.6%) (Fig. 7). These species were mostly used to cure the digestive problems like diarrhea, dysentery, indigestion, stomach-ache and gastrointestinal pain, etc. High FL of a species reflects extensive use of a specific plant species to treat a specific disease dominant in area [13, 17, 114]. Species with high FL value are important model plants which can be subjected to further pharmacological studies [20, 44, 118]. Some other studies in literature also recoded high fidelity level for species used to cure digestive problems [13, 114, 119]. The species with low FL values were not well known by the natives in term of ethnomedicinal knowledge. This forecast that may be in upcoming generation the ethnobotanical knowledge about these plant species may be completely depleted [44, 114, 120] (Table 4).

**Table 4:** Jaccard index comparing the present study with previous articles

Area	SY	NRPs	NPSU	NPDU	TSCBA	SEAA	SESA	PPSU	PPDU	JI	C
From Azad Jammu & Kashmir											
Toil peer (AJK), Pakistan	2017	121	27	18	45	76	105	22.3	14.8	33.08	[41]
Perl valley (AJK), Pakistan	2017	136	29	21	50	86	100	21.3	15.4	36.7	[47]
Neelum Valley (AJK), Pakistan	2011	40	2	7	9	31	141	5	17.5	5.2	[64]
Kotli, AJK, Pakistan	2017	202	21	19	40	162	110	10.3	9.4	17.2	[67]
Bhimber (AJK), Pakistan	2011	38	3	8	11	27	139	7.8	21.05	7.09	[70]
Khahuta (AJK), Pakistan	2013	45	5	9	14	31	136	11.1	20	9.15	[66]
Muzaffarabad (AJK), Pakistan	2014	52	5	13	18	34	132	9.61	25	12.1	[74]
Kotli (AJK), Pakistan	2019	80	11	24	35	45	105	13.7	30	30.4	[40]
Sharda, Neelum Valley (AJK), Pakistan	2012	39	2	9	11	28	139	5.1	23	7.05	[32]
From KPK											
Kabal valley (KPK), Pakistan	2015	45	2	10	12	33	138	4.4	22.2	7.54	[65]
Skardu valley, Karakoram-Himalayan range, Pakistan	2014	50	1	4	5	45	145	2	8	2.7	[39]
Ayubia National Park, Abbottabad, Pakistan	2006	21	3	4	7	14	143	14.2	19.04	4.66	[68]
Tormik valley, Baltistan, Pakistan	2015	63	0	3	3	60	147	0	4.76	1.47	[75]
Northern Pakistani Afghan borders	2018	92	2	18	20	72	148	2.17	19.5	10	[76]
Malakand KPK, Pakistan	2018	25	2	7	9	16	141	8	28	6.08	[77]
Mohmand Agency (FATA), Pakistan	2018	64	1	14	15	49	135	1.56	21.8	8.87	[14]
From other areas of Pakistan											
Wazirabad, Punjab, Pakistan	2018	31	0	4	4	27	146	0	12.9	2.36	[117]
Chenab, Punjab, Pakistan	2019	129	3	24	27	102	123	2.32	18.6	13.6	[78]
Dera Ghazi Khan, Punjab, Pakistan	2012	66	7	9	16	50	134	10.6	13.6	9.52	[69]
From rest of world											
Sikles area, Nepal	2015	42	2	5	7	35	143	4.76	11.9	4.09	[71]
Sangli, Maharashtra, India	2015	21	0	3	3	18	147	0	14.2	1.85	[72]
Kunama ethnic group in Northern Ethiopia	2015	115	1	3	4	111	146	0.86	2.6	1.58	[73] *al.,* 2015

*SY* study year, number of reported plant species; *NPSU* number of plants with similar uses; *NPDU* number of plants with different uses; *TSCBA* total species common in both area; *SEAA* Species enlisted in aligned areas; *SESA* species enlisted only in study area; *PPSU* percentage of plant with similar uses; *PPDU* percentage of plant with different uses; *JI* Jaccard index; *C* citation

### Novel uses

In this study, we compared our results with 22 published papers from adjoining and areas with similar vegetation across Pakistan and world. The highest values for the Jaccard Index (JI) were result of the studies published by Amjad et al. [41] and Shaheen et al. [47] on Toil peer (AJK) and Perl valley respectively. The least value for JI was found in the studies of Jadhava et al. [72] on Sangli, Maharashtra, India, and Gidey et al. [73] on the Kunama ethnic group in Northern Ethiopia. High similarity reflects similar culture, traditions vegetation, and geography among the areas along with high level of cross-cultural exchange of traditional knowledge among the community while high differences or least value of JI reflects that areas do not share common cultural values. Further, the ethno-ecological knowledge is often specifically influenced by origin and culture of indigenous communities.

The comparative study of current findings with reported research revealed some novel uses which were not reported earlier from this region. These included the use of the extract of the whole plant of *Crepis multicaulis* and *Maytenus nemorosa* to treat eye infections. An extract of the aerial parts of *Swertia cordata* was used to treat hepatic disorders. Leaves of *Cotoneaster racemiflora* were used to stop bleeding and pus. The root extract of *Spiraea canescens* is was to as enema to treat venereal conditions. A bark infusion of *Dichanthium annulatum* was used to cure cough. A pasted based on the whole plant of *Polygonum ramosissimum* was used to treat urinary tract infections. The seeds of *Persicaria maculosa* were used in powdered form to treat cholera.

### Threats to medicinal plants and indigenous knowledge

The majority of the local inhabitant in the rural areas of Harighal are illiterate and their main source of income are agriculture and livestock. Some of them collect medicinal plants and sell them at very low prices to local herb sellers. The herbal sellers export herbs to pharmaceutical companies. Over-exploitation of medicinal plant species by untrained collectors, e.g., uprooting of medicinal plants, forest fires, deforestation, over-grazing, and urbanization, are contributing significantly toward the decline of medicinal plant species of the study area, and may finally lead to their extinction. Therefore, authorities should take strict control over protection, conservation, and sustainable utilization of economic plants of the study area. Furthermore, universities, agriculture extension department, and local management may contribute significantly to promote the cultivation of medicinal plants in the area; this will definitely improve the socioeconomic condition of local people of the area.

The traditional practices are highly affected by exposure to modern pharmaceuticals and changing lifestyles. The traditional knowledge about medicinal plants in the study area is gradually declining because this knowledge is now mainly restricted to the older members of the community members which are passing away. The younger generation is not interested in learning about traditional plant use, and makes more use of allopathic medicine. The traditional health practitioners (*Hakeems*) have profound traditional knowledge, but many are not willing to share it with other people. These factors may lead to the erosion of traditional medicinal knowledge among the rural communities of area.

## Conclusion

This study is the first to report the traditional uses of indigenous medicinal plants from the remote areas of tehsil Harighal, Bagh. The documented data reflect that local people are still highly dependent on medicinal plants for treating various diseases, as public health facilities are hard to reach, and still have a large knowledge of medicinal plants. The traditional knowledge is mainly in the hand of elder people and health practitioners (hakims), but the young generation is not much interested in herbal recipes. This lack of interest, as well as impacts like overgrazing, deforestation, and soil erosion, are reducing the medicinal flora in the area, and strategies related to resource conservation and further ethnobotanical and pharmacological research are highly recommended for the conservation of this precious treasure.

## Supplementary information

**Additional file 1:.** Appendix I: Cultivation status and endemism of medicinal flora of Tehsil Harighal.

**Additional file 2:.** Appendix II: Emic and etic use reports of medicinal flora of Harighal.

## References

[1] Arshad M, Ahmad M, Ahmed E, Saboor A, Abbas A, Sadiq S (2014). An ethnobiological study in Kala Chitta hills of Pothwar region, Pakistan: multinomial logit specification. J Ethnobiol Ethnomed.

[2] Ford RI (1978). The nature and status of ethnobotany.

[3] Verpoorte R, Choi YH, Kim HK (2005). Ethnopharmacology and systems biology: a perfect holistic match. J Ethnopharmacol.

[4] Silva FS, Ramos MA, Hanazaki N, UPd A (2011). Dynamics of traditional knowledge of medicinal plants in a rural community in the Brazilian semi-arid region. Revista Brasileira de Farmacognosia.

[5] Cox PA (2000). Will tribal knowledge survive the millennium?. Science.

[6] Khan MPZ, Ahmad M, Zafar M, Sultana S, Ali MI, Sun H (2015). Ethnomedicinal uses of edible wild fruits (EWFs) in Swat Valley, Northern Pakistan. J Ethnopharmacol.

[7] Shabir A, Naveed I, Uneeza J, Noor U, Hina J, Farhat Y (2017). Ethno botanical Wisdom of Inhabitant of Devi Galli Azad Kashmir. Biomedcal J Sci Technol Res.

[8] Shinwari ZK (2010). Medicinal plants research in Pakistan. J Med Plants Res.

[9] Nasir S, Ahmed J, Asrar M (2014). Medicinal plants: a promising resource for poverty alleviation in the milieu of Swat. FUUAST J Biol.

[10] Khatun MA, Harun-Or-Rashid M, Rahmatullah M (2011). Scientific validation of eight medicinal plants used in traditional medicinal systems of Malaysia: a review. American-Eurasian J Sustainable Agriculture.

[11] Shi Q, Li L, Huo C, Zhang M, Wang Y (2010). Study on natural medicinal chemistry and new drug development. Zhongcaoyao - Chinese Traditional Herbal Drugs.

[12] Anna L (1990). Plants for people.

[13] Srithi K, Balslev H, Wangpakapattanawong P, Srisanga P, Trisonthi C (2009). Medicinal plant knowledge and its erosion among the Mien (Yao) in northern Thailand. J Ethnopharmacol.

[14] Aziz MA, Adnan M, Khan AH, Shahat AA, Al-Said MS, Ullah R (2018). Traditional uses of medicinal plants practiced by the indigenous communities at Mohmand Agency, FATA, Pakistan. J Ethnobiol Ethnomed.

[15] Heinrich M, Kufer J, Leonti M, Pardo-de-Santayana M (2006). Ethnobotany and ethnopharmacology—Interdisciplinary links with the historical sciences. J Ethnopharmacol.

[16] Kassaye KD, Amberbir A, Getachew B, Mussema Y (2006). A historical overview of traditional medicine practices and policy in Ethiopia. Ethiopian J Health Development.

[17] Bibi T, Ahmad M, Tareen RB, Tareen NM, Jabeen R, Rehman SU, Zafar M (2014). Ethnobotany medicinal Plants in district Mastung of Balochistan province-Pakistan. J Ethnopharmacol.

[18] Veeresham C (2012). Natural products derived from plants as a source of drugs. J Advanc Pharmaceutical Technol Res.

[19] Mahdi JG (2010). Medicinal potential of willow: A chemical perspective of aspirin discovery. J Saudi Chemical Soc.

[20] Kayani S, Ahmad M, Zafar M, Sultana S, Khan MPZ, Ashraf MA, Hussain J, Yaseen G (2014). Ethnobotanical uses of medicinal plants for respiratory disorders among the inhabitants of Gallies–Abbottabad, Northern Pakistan. J Ethnopharmacol.

[21] Ahmad A, Ali A, Basit A (2019). Ethnomedicinal study of various plants in lone valley, district Chitral, KPK, Pakistan. J Med Plants.

[22] Yaseen G, Ahmad M, Shinwari S, Potter D, Zafar M, Zhang G, Shinwari ZK, Sultana S (2019). Medicinal plants diversity used for livelihood of public health in desert and arid regions of Sindh, Pakistan. Pakistan J Botany.

[23] Pieroni A (2008). Local plant resources in the ethnobotany of Theth, a village in the Northern Albanian Alps. Genetic Resources and Crop Evolution.

[24] Ankli A, Sticher O, Heinrich M (1999). Medical ethnobotany of the Yucatec Maya: healers’ consensus as a quantitative criterion. Econ Botany.

[25] Heinrich M, Gibbons S (2001). Ethnopharmacology in drug discovery: an analysis of its role and potential contribution. J Pharmacy Pharmacol.

[26] Malik AY, Singh D (2019). Ethnobotanical and ethnoveterinary importance of plants of scrub areas of Dachigam national park, Jammu and Kashmir, India. Asian J Pharmaceutical Clin Res.

[27] Vačkář D, ten Brink B, Loh J, Baillie JE, Reyers B (2012). Review of multispecies indices for monitoring human impacts on biodiversity. Ecological Indicators.

[28] Quave CL, Pieroni A (2015). A reservoir of ethnobotanical knowledge informs resilient food security and health strategies in the Balkans. Nature Plants.

[29] Adnan M, Ullah I, Tariq A, Murad W, Azizullah A, Khan AL, Ali N (2014). Ethnomedicine use in the war affected region of northwest Pakistan. J Ethnobiol Ethnomedicine.

[30] Amiri MS, Joharchi MR (2013). Ethnobotanical investigation of traditional medicinal plants commercialized in the markets of Mashhad, Iran. Avicenna J Phytomed.

[31] Vitalini S, Iriti M, Puricelli C, Ciuchi D, Segale A, Fico G (2013). Traditional knowledge on medicinal and food plants used in Val San Giacomo (Sondrio, Italy)—an alpine ethnobotanical study. J Ethnopharmacol.

[32] Ahmad KS, Qureshi R, Hameed M, Ahmad F, Nawaz T. Conservation assessment and medicinal importance of some plants resources from Sharda, Neelum Valley, Azad Jammu and Kashmir Pakistan. Int J Agricultural Biol 2012; 14(6):997-1000.

[33] Tetik F, Civelek S, Cakilcioglu U (2013). Traditional uses of some medicinal plants in Malatya (Turkey). J Ethnopharmacol.

[34] Baydoun S, Chalak L, Dalleh H, Arnold N (2015). Ethnopharmacological survey of medicinal plants used in traditional medicine by the communities of Mount Hermon, Lebanon. J Ethnopharmacol.

[35] Ali S (2008). Significance of flora with special reference to Pakistan. Pakistan J Botany.

[36] Ijaz F, Iqbal Z, Alam J, Khan SM, Afzal A, Rahman I, Afzal M, Islam M, Sohail M. Ethno medicinal study upon folk recipes against various human diseases in Sarban Hills, Abbottabad, Pakistan. *World Journal of Zoology* 2015; 10(1):41-46.

[37] Ali H, Qaiser M (2009). The ethnobotany of Chitral valley, Pakistan with particular reference to medicinal plants. Pakistan J Botany.

[38] Shinwari ZK, Qaiser M (2011). Efforts on conservation and sustainable use of medicinal plants of Pakistan. Pakistan J Botany.

[39] Bano A, Ahmad M, Saboor A, Hadda BT, Zafar M, Sultana S, Ashra MA (2014). Quantitative ethnomedicinal study of plants used in the Skardu valley at high altitude of Karakoram-Himalayan range Pakistan. J Ethnobiol Ethnomed.

[40] Qaseem MF, Qureshi R, Amjad MS, Waseem M, Sajid A (2019). Ethnobotanical evaluation of tridational medicinal plants among thre rular communities of Goi and Dhanwa union council, District Kotli, Azad Jammu & Kashmir. Appl Ecol Environm Res.

[41] Amjad MS, Faisal Qaeem M, Ahmad I, Khan SU, Chaudhari SK, Malik NZ, Shaheen H, Khan AM (2017). Descriptive study of plant resources in the context of the ethnomedicinal relevance of indigenous flora: A case study from Toli Peer National Park, Azad Jammu and Kashmir, Pakistan. PlosOne.

[42] Ullah M, Khan MU, Mahmood A, Malik RN, Hussain M, Wazir SM, Daud M, Shinwari ZK. An ethnobotanical survey of indigenous medicinal plants in Wana district south Waziristan agency, Pakistan. J Ethnopharmacol 2013, 150(3):918-924. 10.1016/j.jep.2013.09.032 24120747

[43] Khan MA, Khan MA, Hussain M, Ghulam GM (2010). An ethnobotanical inventory of Himalayan region poonch valley azad kashmir (Pakistan). Ethnobotany Res Applications.

[44] Ahmad KS, Hamid AF, Nawaz F, Hameed M, Ahmad F, Deng J, Mahroof S (2017). Ethnopharmacological studies of indigenous plants in Kel village, Neelum Valley, Azad Kashmir. J Ethbiol Ethmed.

[45] Anonymous (2007). AJK at a Glance. – Pakistan Planning & Development Department. Government of Azad Jammu and Kashmir, Muzaffarabad.

[46] Ahlaq R, Amjad MS, Qaseem MF, Fatima S, Chaudhari SK, Khan AM, Khan S, Malik NZ, Gardazi SMH, Bibi A (2018). Saboon. Species diversity and vegetation structure from different climatic zones of tehsil Harighel, Bagh, Azad Kashmir, Pakistan analyzed through multivariate techniques. Appl Ecol Environmen Res.

[47] Shaheen H, Qaseem MF, Amjad MS, Bruschi P (2017). Exploration of ethno-medicinal knowledge among rural communities of Pearl Valley; Rawalakot, District Poonch Azad Jammu and Kashmir. PlosOne.

[48] Edwards S, Nebel S, Heinrich M (2005). Questionnaire surveys: methodological and epistemological problems for field-based ethnopharmacologists. J Ethnopharmacol.

[49] Kadam P, Bhalerao S (2010). Sample size calculation. Int J Ayurveda Res.

[50] Jain SK (1977). Handbook of field and herbarium methods.

[51] Nasir E, Ali S, Stewart RR (1972). Flora of West Pakistan: an annotated catalogue of the vascular plants of West Pakistan and Kashmir: Fakhri.

[52] Nasir YJ, Ali S. Flora of Pakistan. Department of Botany, University of Karachi; National Herbarium; 1994-2010.

[53] Chase MW, Christenhusz M, Fay M, Byng J, Judd WS, Soltis D, Mabberley D, Sennikov A, Soltis PS, Stevens PF (2016). An update of the Angiosperm Phylogeny Group classification for the orders and families of flowering plants: APG IV. Botanical J Linnean Soc.

[54] Gardens RB, Kew MBG (2013). The Plant List, Version 1.1*.* Recuperado el.

[55] Staub PO, Geck MS, Weckerle CS, Casu L, Leonti M (2015). Classifying diseases and remedies in ethnomedicine and ethnopharmacology. Journal of Ethnopharmacology.

[56] Heinrich M, Ankli A, Frei B, Weimann C, Sticher O (1998). Medicinal plants in Mexico: Healers' consensus and cultural importance. Soci. Sci & Med..

[57] Vijayakumar J, Yabesh M, Prabhu S, Manikandanz R, Muralidharan B. Quantitative ethnomedicinalstudy of plants used in the Nelliyampathy hills of Kerala, India*.* J. Ethpharmacol. 2015;161:238-254. 10.1016/j.jep.2014.12.006 25529616

[58] Ugulu I, Baslar S, Yorek N, Dogan Y (2009). The investigation and quantitative ethnobotanical evaluation of medicinal plants used around Izmir province, Turkey. Journal of Medicinal plants research..

[59] Alexiades MN, Sheldon JW (1996). Selected guidelines for ethnobotanical research: a field manual.

[60] Gonza TM, Casares RPM, Sanchez RCP, Ramiro GJM, Molero MJ, Pieroni A (2008). Medicinal plants in the Mediterranean area: synthesis of the results of the project RUBIA. Journal of Ethnopharmacoogyl.

[61] Umair M, Altaf M, Abbasi AM. An ethnobotanical survey of indigenousmedicinal plants in Hafizabad district, Punjab- Pakistan. *PlosOne*, 2017; 12(6):e0177912. 10.1371/journal.pone.0177912 PMC5456064 28574986

[62] Giday M, Asfaw Z, Woldu Z (2009). Medicinal plants of the Meinit ethnic group of Ethiopia: an ethnobotanical study. Journal of Ethnopharmacology.

[63] Tugume P, Esezah KK, Buyinza M, Namaalwa J, Kamatenesi M, Mucunguzi P, Kalema J. Ethnobotanical survey of medicinal plant species used by communities around Mabira Central Forest Reserve, Uganda. *Journal of Ethnobiology and Ethnomedicine* 2010; 12(5). 10.1186/s13002-015-0077-4 PMC4712608 26762159

[64] Mahmood A, Malik RN, Shinwari ZK, Mahmood A (2011). Ethnobotanical survey of plants from Neelum, Azad Jammu and Kashmir, Pakistan. Pakistan J Botany.

[65] Khan S, Din NU, Sohail I, Rahman FI, Iqbal Z, Ali Z (2015). Ethnobotanical study of some medicinal plants of Tehsil Kabal, District Swat, KP. Medicinal Aromatic Plants.

[66] Ch MI, Ahmed F, Maqbool M, Hussain T (2013). Ethnomedicinal inventory of flora of maradori valley, district forward Khahuta, Azad Kashmir, Pakistan. Am J Res Commun.

[67] Amjad MS, Arshad M, Saboor A, Page S, Chaudhari SK (2017). Ethnobotanical profiling of the medicinal flora of Kotli, Azad Jammu and Kashmir, Pakistan: Empirical reflections on multinomial logit specifications. Asian Pacific J Tropical Med.

[68] Gilani SA, Qureshi RA, Gilani SJ (2006). Indigenous uses of some important ethnomedicinal herbs of Ayubia National Park, Abbottabad, Pakistan. Ethnobotanical Leaflets.

[69] Gulshan AB, Dasti AA, Hussain S, Atta MI, Amin-ud-Din M (2012). Indigenous uses of medicinal plants in rural areas of Dera Ghazi Khan, Punjab, Pakistan. ARPN J Agricultural Biological Sci.

[70] Mahmood A, Mahmood A, Shaheen H, Qureshi RA, Sangi Y, Gilani SA (2011). Ethno medicinal survey of plants from district Bhimber Azad Jammu and Kashmir, Pakistan. J Med Plants Res.

[71] Rana SK, Oli PS, Rana HK (2015). Traditional botanical knowledge (TBK) on the use of medicinal plants in Sikles area, Nepal. Asian J Plant Sci Res.

[72] Jadhav RR (2015). Ethnobotanical and ethnomedicinal survey of Kadegaon Tahsil, Sangli (Maharashtra) India. J Medicinal Plants Stud.

[73] Gidey M, Beyene T, Signorini MA, Bruschi P, Yirga G (2015). Traditional medicinal plants used by Kunama ethnic group in Northern Ethiopia. J Medicinal Plants Res.

[74] Dar EM (2014). Ethnobotanical uses of plants of Lawat district Muzaffarabad, Azad Jammu & Kashmir. Asian J Plant Sci.

[75] Khan SW, Abbas Q, Hassan SN, Khan H, Hussain A (2015). Medicinal plants of Turmic Valley (Central Karakoram National Park), Gilgit-Baltistan, Pakistan. J Bioresource Management.

[76] Hussain W, Badshah L, Ullah M, Ali M, Ali A, Hussain F (2018). Quantitative study of medicinal plants used by the communities residing in Koh-e-Safaid Range, northern Pakistani-Afghan borders. J Ethnobiol Ethnomed.

[77] Ullah S, Bibi S. Ethnobotanical survey of medicinal plants of Musamina District Malakand Khyber Pukhtoonkhwa, Pakistan. Academic J Med Plants. 2018;6(6).

[78] Umair M, Altaf M, Bussmann RW, Abbasi AM (2019). Ethnomedicinal uses of the local flora in Chenab riverine area, Punjab province Pakistan. J Ethnobiol Ethnomed.

[79] Akhtar N, Rashid A, Murad W, Bergmeier E (2013). Diversity and use of ethno-medicinal plants in the region of Swat, North Pakistan. J Ethnobiol Ethnomed.

[80] Kadir MF, Sayeed MSB, Setu NI, Mostafa A, Mia M (2014). Ethnopharmacological survey of medicinal plants used by traditional health practitioners in Thanchi, Bandarban Hill Tracts, Bangladesh. J Ethnopharmacol.

[81] Jan G, Khan MA, Farhatullah JF, Ahmad M, Jan M, Zafar M (2011). Ethnobotanical studies on some useful plants of Dir Kohistan valleys, KPK, Pakistan. Pakistan J Botany.

[82] Miller NJ, Ruiz-Larrea MB (2002). Flavonoids and other plant phenols in the diet: their significance as antioxidants. J Nutritional Environmen Med.

[83] Wickens GE, Field DV, Goodin JR (2012). Plants for Arid Lands: Proceedings of the Kew International Conference on Economic Plants for Arid Lands Held in the Jodrell Laboratory, Royal Botanic Gardens, Kew, England, 23–27 July 1984: Springer Science & Business Media.

[84] Milliken W, Albert B, Gomez GG (1999). Yanomami: a forest people: Royal Botanic Gardens, Kew.

[85] Savoia D (2012). Plant-derived antimicrobial compounds: alternatives to antibiotics. Future Microbiol.

[86] Bradacs G, Heilmann J, Weckerle CS (2011). Medicinal plant use in Vanuatu: a comparative ethnobotanical study of three islands. J Ethnopharmacol.

[87] Leto C, Tuttolomondo T, La Bella S, Licata M (2013). Ethnobotanical study in the Madonie Regional Park (Central Sicily, Italy)—Medicinal use of wild shrub and herbaceous plant species. J Ethnopharmacol.

[88] Cornara L, La Rocca A, Marsili S, Mariotti M (2009). Traditional uses of plants in the Eastern Riviera (Liguria, Italy). J Ethnopharmacol.

[89] Neves JM, Matos C, Moutinho C, Queiroz G, Gomes LR (2009). Ethnopharmacological notes about ancient uses of medicinal plants in Trás-os-Montes (northern of Portugal). J Ethnopharmacol.

[90] Khan SM, Page S, Ahmad H, Shaheen H, Ullah Z, Ahmad M, Harper DM (2013). Medicinal flora and ethnoecological knowledge in the Naran Valley, Western Himalaya, Pakistan. J Ethnobiol Ethnomed.

[91] Zheng X, Xing F (2009). Ethnobotanical study on medicinal plants around Mt. Yinggeling, Hainan Island, China. J Ethnopharmacol.

[92] Panyaphu K, Van On T, Sirisa-Ard P, Srisa-Nga P, ChansaKaow S, Nathakarnkitkul S (2011). Medicinal plants of the Mien (Yao) in Northern Thailand and their potential value in the primary healthcare of postpartum women. J Ethnopharmacol.

[93] Ghimire SK, Gimenez O, Pradel R, McKey D, Aumeeruddy-Thomas Y (2008). Demographic variation and population viability in a threatened Himalayan medicinal and aromatic herb Nardostachys grandiflora: matrix modelling of harvesting effects in two contrasting habitats. J Appl Ecol.

[94] Giday M, Asfaw Z, Elmqvist T, Woldu Z (2003). An ethnobotanical study of medicinal plants used by the Zay people in Ethiopia. J Ethnopharmacol.

[95] Ahmad M, Sultana S, Fazl-i-Hadi S, Ben Hadda T, Rashid S, Zafar M, Khan MA, Khan Ahmad M, Sultana S, Fazl-i-Hadi S, Ben Hadda T, Rashid S, Zafar M, Khan MA, Khan MPZ, Yaseen G (2014). An Ethnobotanical study of Medicinal Plants in high mountainous region of Chail valley (District Swat-Pakistan). J Ethnobiol Ethnomed.

[96] Inta A, Trisonthi P, Trisonthi C (2013). Analysis of traditional knowledge in medicinal plants used by Yuan in Thailand. J Ethnopharmacol.

[97] El Amri J, El Badaoui K, Zair T, Bouharb H, Chakir S, Alaoui TEM (2015). Ethnobotanical study of medicinal plants in the region El Hajeb (central Morocco). J Res Biol.

[98] Zhang JL, Cui M, He Y, Yu HL, Guo DA (2005). Chemical fingerprint and metabolic fingerprint analysis of Danshen injection by HPLC–UV and HPLC–MS methods. J Pharmaceutical Biomedical Analysis.

[99] JA. An ethnobotanical study of medicinal plants used by tribal and native people of Madhupur forest area Bangladesh. J Ethnopharmacol 2014; 151(2):921-930. 10.1016/j.jep.2013.11.056 24342778

[100] Sanon S, Ollivier E, Azas N, Mahiou V, Gasquet M, Ouattara C, Nebie I, Traore A, Esposito F, Balansard G (2003). Ethnobotanical survey and in vitro antiplasmodial activity of plants used in traditional medicine in Burkina Faso. J Ethnopharmacol.

[101] Siew YY, Zareisedehizadeh S, Seetoh WG, Neo SY, Tan CH, Koh HL (2014). Ethnobotanical survey of usage of fresh medicinal plants in Singapore. J Ethnopharmacol.

[102] Uddin MZ, Hassan MA (2014). Determination of informant consensus factor of ethnomedicinal plants used in Kalenga forest, Bangladesh. Bangladesh J Plant Taxonomy.

[103] Heinrich M, Edwards S, Moerman DE, Leonti M (2009). Ethnopharmacological field studies: a critical assessment of their conceptual basis and methods. J Ethnopharmacol.

[104] Ghorbani A, Langenberger G, Feng L, Sauerborn J (2011). Ethnobotanical study of medicinal plants utilised by Hani ethnicity in Naban river watershed national nature reserve, Yunnan, China. J Ethnopharmacol.

[105] Miraldi E, Ferri S, Mostaghimi V (2001). Botanical drugs and preparations in the traditional medicine of West Azerbaijan (Iran). J Ethnopharmacol.

[106] Mosaddegh M, Naghibi F, Moazzeni H, Pirani A, Esmaeili S (2012). Ethnobotanical survey of herbal remedies traditionally used in Kohghiluyeh va Boyer Ahmad province of Iran. J Ethnopharmacol.

[107] Tangjitman K, Wongsawad C, Kamwong K, Sukkho T, Trisonthi C (2015). Ethnomedicinal plants used for digestive system disorders by the Karen of northern Thailand. J Ethnobiol Ethnomed.

[108] Malla B, Gauchan DP, Chhetri RB (2015). An ethnobotanical study of medicinal plants used by ethnic people in Parbat district of western Nepal. J Ethnopharmacol.

[109] Murad W, Azizullah A, Adnan M, Tariq A, Khan KU, Waheed S, Ahmad A (2013). Ethnobotanical assessment of plant resources of Banda Daud Shah, district Karak, Pakistan. J Ehnobiol Ethnomed.

[110] Adzu B, Amos S, Amizan M, Gamaniel K (2003). Evaluation of the antidiarrhoeal effects of Zizyphus spina-christi stem bark in rats. ActaTtropica.

[111] Schlage C, Mabula C, Mahunnah R (2000). Heinrich. Medicinal plants of the Washambaa (Tanzania): documentation and ethnopharmacological evaluation. Plant Biol.

[112] Mukherjee PK, Nema NK, Venkatesh P, Debnath PK (2012). Changing scenario for promotion and development of Ayurveda±way forward. J Ethnopharmacl.

[113] Trotter R, Logan M, Trotter R, Logan M. Informant consensus: a new approach for identifying potentially effective medicinal plants. In: Etkin NL, editor. Plants and Indigenous Medicine and Diet - Behavioral Approaches 1986: Taylor and Francis.

[114] Farooq A, Amjad MS, Ahmad K, Altaf M, Umair M, Abbasi AM (2019). Ethnomedicinal knowledge of the rural communities of Dhirkot, Azad Jammu and Kashmir, Pakistan. J Ethnobiol Ethnomed.

[115] Camou-Guerrero A, Reyes-García V, Martínez-Ramos M, Casas A (2008). Knowledge and use value of plant species in a Rarámuri community: a gender perspective for conservation. Human Ecol.

[116] Albuquerque UP, Lucena RF, Monteiro JM, Florentino AT, Cecília de Fátima C (2006). Evaluating two quantitative ethnobotanical techniques. Ethnobotany Res Appl.

[117] Noreen F, Tamoor M, Adil M, Mushtaq U, Nisa Q (2018). Data of ethnomedicinal plants in Wazirabad, District Gujranwala, Punjab. Pakistan J Pharma Care Health Syst.

[118] Hassan-Abdallah A, Merito A, Hassan S, Aboubaker D, Djama M, Asfaw Z, Kelbessa E (2013). Medicinal plants and their uses by the people in the Region of Randa, Djibouti. J Ethnopharmacol.

[119] Lulekal E, Kelbessa E, Bekele T, Yineger H (2008). An ethnobotanical study of medicinal plants in Mana Angetu District, southeastern Ethiopia. J Ethnobiol Ethnomedicine.

[120] Yineger H, Yewhalaw D, Teketay D (2008). Ethnomedicinal plant knowledge and practice of the Oromo ethnic group in southwestern Ethiopia. J Ethnobiol Ethnomed.

