# Supplementary material for: Ethnobotanical survey of the medicinal flora of Harighal, Azad Jammu & Kashmir, Pakistan
Source: J Ethnobiol Ethnomed. 2020 Oct 27;16:65. doi: 10.1186/s13002-020-00417-w (PMC7590686; doi:10.1186/s13002-020-00417-w)
Supplement: Supplementary file 1 — Additional file 1:. Appendix I: Cultivation status and endemism of medicinal flora of Tehsil Harighal. [file 13002_2020_417_MOESM1_ESM.docx]

**Appendix I: Cultivation status and endemism of medicinal flora of Tehsil Harighal**

| **Scientific name** | **Cultivation status** | **Indigenous/ exotic** |
| --- | --- | --- |
| *Abutilon ramosum* | W | I |
| *Acacia modesta* | W | I |
| *Acacia nilotica* | W/C | I |
| *Achillea millefolium* | W | E |
| *Achyranthes aspera* | W | E |
| *Adiantum tenerum* | W | E |
| *Aesculus indica* | W | I |
| *Ajuga bracteosa* | W | I |
| *Ailanthus altissima* | W | E |
| *Allium grifﬁthianum* | W | I |
| *Alternanthera pungens* | W | E |
| *Amaranthus spinosus* | W | E |
| *Amaranthus viridis* | W | E |
| *Anagallis arvensis* | W | E |
| *Anaphalis adnata* | W | E |
| *Androsace rotundifolia* | W | I |
| *Angelica glauca* | W | I |
| *Artemisia vulgaris* | W | I |
| *Asplenium dalhousiae* | W | E |
| *Astragalus canadensis* | W | E |
| *Bauhinia variegata* | W/C | I |
| *Berberis lycium* | W | I |
| *Bidens biternata* | W | I |
| *Callicarpa mycrophylla* | W | I |
| *Cannabis sativa* | W | E |
| *Capsella bursa-pastoris* | W | E |
| *Carissa opaca* | W | I |
| *Cirsium vulgare* | W | E |
| *Chenopodium album* | W | E |
| *Chrysopogon serrulatus* | W | I |
| *Clematis grata* | W | I |
| *Commelina benghalensis* | W | I |
| *Convolvulus arvensis* | W | E |
| *Conyza canadensis* | W | E |
| *Cornus macrophylla* | W | I |
| *Cotoneaster racemiflora* | W | I |
| *Crepis multicaulis* | W | E |
| *Crotalaria juncea* | W | I |
| *Cuscuta reflexa* | W | I |
| *Cynodon dactylon* | W | I |
| *Cynoglossum lanceolatum* | W | E |
| *Cyperus rotundus* | W | E |
| *Dactylis glomerata* | W | E |
| *Dalbergia sissoo* | W | I |
| *Debregeasia salicifolia* | W | I |
| *Desmodium elegans* | W | I |
| *Dichanthium annulalum* | W | I |
| *Dicliptera bupleuroides* | W | I |
| *Digitalis ciliata* | W | I |
| *Dodonaea viscosa* | W | E |
| *Dryopteris filix-mas* | W | E |
| *Duchesnea indica* | W | I |
| *Echinochloa colona* | W | E |
| *Elaeagnus umbellata* | W | E |
| *Eleusine indica* | W | E |
| *Eriobotrya japonica* | C | E |
| *Eucalyptus* *camaldulensis* | W | E |
| *Euphorbia helioscopia* | W | E |
| *Ficus carica* | W | E |
| *Ficus palmate* | W | I |
| *Fragaria nubicola* | W | E |
| *Galium aparine* | W | E |
| *Geranium rotundifolium* | W | E |
| *Gerbera gossypina* | W | I |
| *Hedera nepalensis* | W | I |
| *Helianthus annuus* | C | E |
| *Heteropogon contortus* | W | I |
| *Impatiens edgeworthii* | W | I |
| *Imperata cylindrica* | W | E |
| *Indigofera linifolia* | W | I |
| *Ipomoea purpurea* | W | E |
| *Jasminum officinale* | W | I |
| *Juglans regia* | W | I |
| *Justicia adhatoda* | W | I |
| *Lathyrus aphaca* | W | I |
| *Launaea procumbens* | W | I |
| *Lespedeza juncea* | W | I |
| *Lolium temulentum* | W | E |
| *Lotus corniculatus* | W | I |
| *Malva parviflora* | W | I |
| *Malvastrum coromandelianum* | W | E |
| *Maytenus nemorosa* | W | I |
| *Medicago polymorpha* | W | E |
| *Melia azadrachta* | W | E |
| *Mentha spicata* | W/C | E |
| *Mentha longifolia* | W | E |
| *Micromeria biflora* | W | I |
| *Mimosa pudica* | W | E |
| *Morus alba* | C | E |
| *Morus nigra* | C | E |
| *Nerium oleander* | W | I |
| *Ocimum sanctum* | W | I |
| *Oenothera rosea* | W | E |
| *Olea ferruginea.* | W | I |
| *Onychium japonicum* | W | E |
| *Origanum vulgare* | W | E |
| *Otostegia limbata* | W | I |
| *Oxalis corniculata* | W | I |
| *Persicaria maculosa* | W | I |
| *Phlomis bracteosa* | W | I |
| *Pinus roxburghii* | W | I |
| *Plantago lanceolata* | W | E |
| *Polygala abyssinica* | W | I |
| *Polygonatum geminiflorum* | W | I |
| *Polygonum ramosissimum* | W | E |
| *Populus nigra* | C | E |
| *Potentilla reptans* | W | E |
| *Prunella vulgaris* | W | E |
| *Prunus armeniaca* | C | E |
| *Prunus domestica* | C | E |
| *Prunus persica* | C | E |
| *Pteris vittata* | W | E |
| *Punica granatum* | W | I |
| *Pyrus malus* | C | I |
| *Pyrus pashia* | W | I |
| *Ranunculus arvensis* | W | E |
| *Rauanculus muricatus* | W | E |
| *Ricinus communis* | W | E |
| *Rosa brunoni* | W | E |
| *Rosa indica* | C | I |
| *Rubus ellipticus* | W | I |
| *Rubus niveus* | W | I |
| *Rumex hastatus* | W | E |
| *Salvia lanata* | W | I |
| *Sarcococca saligna* | W | I |
| *Setaria pumila* | W | E |
| *Silybum marianum* | W | E |
| *Solanum villosum* | W | E |
| *Sonchus oleraceus* | W | I |
| *Spiraea canescens* | W | I |
| *Swertia cordata* | W | I |
| *Tagetes minuta* | W | E |
| *Taraxacum officinale* | W | I |
| *Thalictrum revolutum* | W | E |
| *Themeda anathera* | W | E |
| *Thymus linearis* | W | E |
| *Torilis japonica* | W | E |
| *Trichodesma indicum* | W | I |
| *Trifolium resupinatum* | W | I |
| *Urtica dioica* | W | E |
| *Valeraina jatamansi* | W | I |
| *Verbascum thapsus* | W | E |
| *Verbena officinalis* | W | E |
| *Veronica laxa* | W | I |
| *Viburnum grandiflorum* | W | I |
| *Vicia sativa* | W | E |
| *Vincetoxicum hirundinaria* | W | I |
| *Viola odorata* | W | I |
| Vitex *agnus-castus* | W | I |
| *Xanthium strumarium* | W | E |
| *Zanthoxylum armatum* | W/C | I |
