# Supplementary material for: Ethnobotanical survey of the medicinal flora of Harighal, Azad Jammu & Kashmir, Pakistan
Source: J Ethnobiol Ethnomed. 2020 Oct 27;16:65. doi: 10.1186/s13002-020-00417-w (PMC7590686; doi:10.1186/s13002-020-00417-w)
Supplement: Supplementary file 2 — Additional file 2:. Appendix II: Emic and etic use reports of medicinal flora of Harighal. [file 13002_2020_417_MOESM2_ESM.docx]

**Appendix II:** Emic and etic use reports of medicinal flora of Harighal

| **Etic use reports** | **Emic use reports** | **Ntax** | **Nur** | **ICF** | **Most Used Plans** |
| --- | --- | --- | --- | --- | --- |
| Mouth-Throat Diseases | Toothache, Sore throat, Gum Infection, Mouth Infection and inflammation | 58 | 132 | 0.56 | *Nerium oleander, Bidens biternata, Cynoglossom lanceolatum, Maytenus nemorosa, Geranium officinale, Juglan sregia, Melia azadrachta, Acacia modesta, Morus alba , Eucalyptus spp, Olea ferruginea, Populous alba, Cerscium vulgare, Plantago lanceolate* |
| Eye and Ear Diseases | Eye Diseases, Earache | 65 | 104 | 0.37 | *Crepis multicaulis, Achyranthes aspera, Pyrus pashia, Rosa indica, Ricinus communis, Amaranthus viridis, Ajuga bracteosa, Setaria pumila* |
| Diabetes | Diabetes | 25 | 52 | 0.52 | *Fragaria nubicola, Clematis grata, Ficus palmate, Melia azadrachta, Swertia cordata, Taraxacum officinale, Launaea procumbens, Diclyptera roxburghiana* |
| Respiratory tract diseases | Cough, Common Cold, Flue, Pneumonia, Mastitis, Lungs disease, Breathing disorders, Asthma, Influenza, Chest Infection | 84 | 225 | 0.62 | *Diclyptera roxburghiana, Adhatoda zeylanica, Angelica glauca, Carissa opaca, Nerium oleander, Achillea millefolium, Xanthium strumarium, Helianthus annus, Capsella bursa-pastoris, Viburnum grandiflorum, Thymus liniaris, Prunella vulgaris, Salvia lanata, Oxalis corniculata,Vicia sativa, Dichanthium annulalum, Eleusine indica* |
| Muscular and Joint Diseases | Joint pain, Rickets, Backache, Muscle pain, Paralysis, Sciatica, Rheumatism, Bone fracture, Knee pain, Body and Joint inflammation, Arthritis, Muscles weakness, Bells' palsy | 33 | 114 | 0.71 | *Carissa opaca, Cerscium vulgare, Gerbera gossypina, Ricinus communis, Prunella vulgaris, Cynodon dactylon, Themeda anathera, Populous alba, Callicarpa mycrophyllus, Aesculus indica, Pyrus malus, Rubus niveus, Dalbergia sissoo, Olea ferouginea* |
| Digestive system and Liver diseases | Dysentery, Indigestion, Diarrhea, Kill insect, Stomach diseases *,* Biliousness , Constipation, Gastric problems, Jaundice, Liver diseases, Motion, Gall stones, Bile disorders, Bladder ailment, Bloody diarrhea, Vomiting, Bowel Complaints, Hepatitis, Stomach pain, Stomach acidity, Liver tonic, Dyspepsia, Liver pain, Peptic ulcer, Motion, Cholera | 45 | 901 | 0.95 | *Mentha viridis, Bauhinia variegate, Punica granatum, Abutilon romosum, Malva palviflora , Malvastrum coromandelianum, Melia azadrachta, Acacia nelotica, Morus alba ,Morus nigra, Ficus palmate, Ficus carica, Oxalis corniculata, Dalbergia sissoo, Lespedeza juncea, Eriobotrya japonica, Rosa indica, Pyrus pashia, Prunus armeniaca,* |
| Skin diseases (Infections, Burns, Blisters, Swelling, Wound healing) | Skin Infection, Wound Healing, Swelling, Blister, Burns, Eczema, Measles, Scabies, Warts, Sunburn, Cuts, inflammation, Bruises, Itching, Skin ulcers, Wound worms, Anti-inflammatory, Boils, Pimples Cracks, Face spots, Dark circles around eyes, Sore between toes | 31 | 337 | 0.91 | *Diclyptera roxburghiana, Alternanthera popua, Achyranthes aspera,Torilis japonica, Carissa opaca, Euphorbia heliscopia, Ricinus communis, Origanum vulgare, Otostegia limbata, Melia azadrachta, Pinus roxburghii, Plantago lanceolate, Verbena officinalis, Verbena officinalis, Adiantum tenerum, Dryopteris filix–mas, Cannabis sativa* |
| Circulatory system diseases | Bleeding, Hypertension, Hemorrhoids, Cardiovascular diseases, | 27 | 54 | 0.50 | *Nerium oleander, Conyza canadensis, Capsella bursa-pastoris, Geranium officinale, Punica granatum, Melia azadrachta, Digitalis ciliate, Rubus elipticus, Rosa brunonni, Eriobotrya japonica,Duchesniaindica, Pyrusmalus* |
| Urinary and Reproductive system diseases | Scanty urination, Menstrual problem, Amenorrhea, Kidney diseases, Menopause complaints, Menorrhagia, Syphilis, Sexual disorders, Abortion, Urinary tract infection, Abortion, Miscarriage | 58 | 132 | 0.56 | *Verbena officinalis, Solanum villosum, Populous alba, Galium aparine, Rubus elipticus,Polygonum ramosissimum,Vicia sativa, Trichodesma indicum, Xanthium strumarium, Silybum marianum , Helianthus annus, Oenothera rosea, Olea ferouginea* |
| Fever | Hay fever, Chronic fever, Antipyretic, Internal fever. | 12 | 91 | 0.87 | *Nerium oleander, Angelica glauca, Xanthium strumarium, Melia azadrachta, Oxalis corniculata, Thalictrum revolutum ,Rubus niveus, Rosa indica, Populous alba* |
| Cancer | Cancer, Breast cancer, Tumor. | 22 | 40 | 0.46 | *Verbascum thapsun, Dactylis glomerata, Digitalis ciliate,Mimosa pudica, Ipomea purpurea* |
| Hair problems | Hair growth, Hair fall, Dandruff. | 15 | 35 | 0.23 | *Cuscuta reflexa, Convolvulus arvensis, Acacia nelotica, Jasminum officinale, Crotalaria juncea* |
| Nervous system diseases | Headache, Migraine, Mental disorders, Epilepsy, Nerve disorders, Coma, Narcotic, Anxiety, Depression, Convulsions, Insomnia | 34 | 79 | 0.57 | *Anagallis arvensis, Juglans regia, Cannabis sativa, Trichodesma indicum* |
| Blood and Lymphatic system diseases | Anemia, Blood purification, Blood clotting. | 15 | 45 | 0.69 | *Ajuga bracteosa, Themeda anathera, Ailanthus altissima, Melia azedarach, Rubus niveus, Oxalis corniculata* |
| Antitode | Snake bite, Scorpion sting, Insect bites. | 9 | 30 | 0.72 | *Amaranthus viridis, Achyranthes aspera, Ailanthus altissima, Fragaria vesca*, *Melia azadarach, Oxalis corniculata, Rubus niveus, Sarcococca saligna* |
| Others | Dropsy (oedema), Gout disease, Body weakness, Hypothermia, Malaria. | 27 | 63 | 0.58 | *Hedera nepalensis, Achillea millefolium, Xanthium strumarium, Capsella bursa-pastoris, Oxalis corniculata, Vicia sativa, Pyrus malus, Zanthoxylum alatum, Populous alba* |
